# *Halszkaraptor escuilliei* and the evolution of the paravian *bauplan*

**DOI:** 10.1038/s41598-019-52867-2

**Published:** 2019-11-11

**Authors:** Chase D. Brownstein

**Affiliations:** Research Associate, Dept. of Collections & Exhibitions, Stamford Museum and Nature Center, Stamford, USA

**Keywords:** Phylogenetics, Palaeontology

## Abstract

The evolution of birds from dinosaurs is a subject that has received great attention among vertebrate paleontologists. Nevertheless, the early evolution of the paravians, the group that contains birds and their closest non-avian dinosaur relatives, remains very poorly known. Even the most basal members of one paravian lineage, the Dromaeosauridae, already show a body plan that differs substantially from their closest non-paravian relatives. Recently, the dromaeosaurid *Halszkaraptor escuilliei* was described from the Cretaceous of Mongolia. *Halszkaraptor* possesses numerous unserrated premaxillary teeth, a platyrostral rostrum with a developed neurovascular system, an elongate neck, bizarrely-proportioned forearms, and a foreword-shifted center of mass, differing markedly from other paravians. A reevaluation of the anatomy, taphonomy, environmental setting, and phylogenetic position of *H*. *escuilliei* based on additional comparisons with other maniraptorans suggests that, rather than indicating it was a semiaquatic piscivore, the body plan of this dinosaur bears features widely distributed among maniraptorans and in some cases intermediate between the conditions in dromaeosaurids and related clades. I find no evidence for a semiaquatic lifestyle in *Halszkaraptor*. A phylogenetic reevaluation of Halszkaraptorinae places it as the sister clade to Unenlagiinae, indicating the bizarre features of unenlagiines previously interpreted as evidence of piscivory may also represent a mosaic of plesiomorphic, derived, and intermediate features. The anatomy of *Halszkaraptor* reveals that dromaeosaurids still possessed many features found in more basal maniraptoran and coelurosaur clades, including some that may have been tied to herbivory. Rather than being a semiaquatic piscavore, *Halszkaraptor* was a basal dromaeosaurid showing transitional features.

## Introduction

The dinosaur lineage including birds and close relatives is known as the Maniraptora^1^ and includes a diverse array of genera. One clade within this group, the paravians, includes birds and the sickle-clawed troodontids and dromaeosaurids. The ancestral maniraptoran body plan seems to have been adapted for omnivory or herbivory, and members of lineages bracketing the paravian stem, including therizinosaurs, oviraptorosaurs, alvarezsaurs, and ornithomimosaurs, shared features like a long neck, an elongate skull with many small teeth or an edentulous jaw with rhamphotheca, a deepened thorax, and a forward-shifted center of mass^1–14^. Notably, many of these traits seem to be related to the development of an omnivorous or herbivorous diet in these clades^1–5,8,11–13,15–19^. However, despite extensive research of the anatomy of both paravians and their maniraptoran relatives, the transition between the body plans of more basal maniraptorans and the specialized, hypercarnivorous one found in dromaeosaurids remains obscure. Like some troodontids and basal birds, dromaeosaurids possessed recurved, serrated, ziphodont teeth, lacked extensive rhamphotheca, bore an enlarged claw on the second digit of the foot, and possessed rather less deepened torsos and less elongate necks than those found in more basal maniraptoran clades^20–24^. Even the most basal members of Dromaeosauridae, the bizarre Gondwanan unenlagiines, possessed a mediolaterally compressed skull, a jaw packed with recurved, ziphodont teeth, a backward-shifted center of mass balanced by a long tail, and an enlarged ‘sickle’ claw on the pes^20,21,25–31^.

Recently, Cau *et al*.^32^ described the dromaeosaurid *Halszkaraptor escuilliei* from the Late Cretaceous deposits of Mongolia and found this new genus of dinosaur and two other Asian dromaeosaurids, *Mahakala omnogovae* and *Hulsanpes perlei*, formed a clade at the base of Dromaeosauridae they named the Halszkaraptorinae. Cau *et al*.^32^. described the bizarre body plan of *Halszkaraptor*, which they suggested was adapted for a semiaquatic, ichthyophagous lifestyle. If this hypothesized ecomorphology for *Halszkaraptor* is correct, it has major implications for the evolution of bird-like dinosaurs, with *H*. *escuilliei* representing the first aquatic non-avian maniraptoran and suggesting that the ancestral lifestyle for dromaeosaurids could be one that took place in the water^32^. Among the many features in *Halszkaraptor* considered to be adaptations for an aquatic lifestyle by Cau *et al*.^32^ are those they considered to represent convergences between *H*. *escuilliei* and semiaquatic non-avian dinosaurs, marine birds, crocodylians, turtles, and marine reptiles.

Given the importance of *Halszkaraptor escuilliei*, a basal dromaeosaurid, for understanding the evolution of the dromaeosaurid body plan and the level of ecomorphological diversification that took place along the dinosaur-bird transition, further evaluation of the anatomy of this species is paramount. Here, I present an extensive reevaluation of the supposed semiaquatic adaptations of *Halszkaraptor* based on detailed comparisons with representatives of clades along the maniraptoran stem and a revised phylogenetic analysis. Despite the apparent aberrancy of the skeleton of *Halszkaraptor*, virtually all of the distinctive features of this taxon Cau *et al*.^32^ suggested were indicators of ichthyophagy and a semiaquatic ecology are widespread among maniraptorans and other bird-like dinosaurs, and many are probably plesiomorphic to Maniraptora or less-inclusive clades. Others seem to represent homoplastic features that neither alone nor together can be considered strong evidence for a unique ecological mode in *Halszkaraptor*. Instead of a being a semiaquatic piscivore, *Halszkaraptor* instead is likely representative of the morphological transition from the ancestral body plan of maniraptorans to the one that characterized dromaeosaurids.

## Results

### Comparative Anatomy of *Halszkaraptor*

*Halszkaraptor* possesses a set of aberrant characteristics that together produce a *bauplan* superficially unlike those of other known paravian theropods^32^. Because *Halszkaraptor* was originally interpreted as a dromaeosaurid paravian, its morphology was differentiated from the other members of that lineage, which are all terrestrial and arboreal^20,21^. However, the anatomy of *Halszkaraptor* was not extensively compared with non-paravian maniraptorans, which ought to be done given the taxon’s basal phylogenetic position within Dromaeosauridae and Paraves at large^32^. Below is a comprehensive review of the comparative anatomy of *Halszkaraptor*, many of which support a critical reassessment of the hypothesis that this taxon was an aquatic piscavore. This reevaluation is based on an extensive review of the literature and firsthand examination of several specimens.

### Rostral neurovasculature

**Figure 1:**
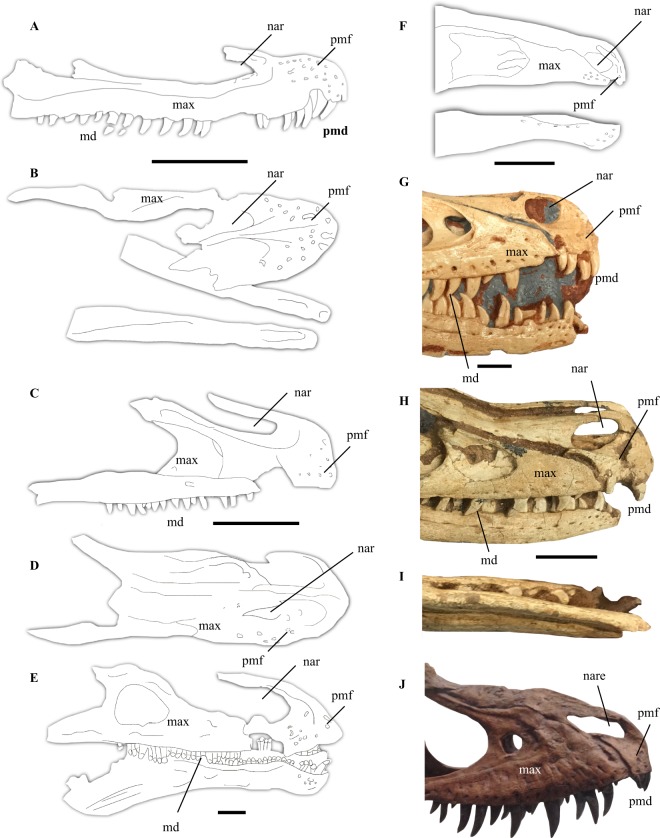
Rostral anatomy of select theropods. Rostrum of *Halszkaraptor* after Cau *et al*.^32^ in lateral (**A**) and dorsal (**B**) views. Rostrum of *Erlikosaurus* after Lautenschlager *et al*.^19^ in (**C**) lateral and dorsal (**D**) views. Rostrum of *Jianchangosaurus* after Pu *et al*.^18^ in (**E**) lateral view. Rostrum of *Harpymimus* after Kobayashi and Barsbold^46^ in (**F**) lateral view. Rostrum of *Tsaagan* (cast) in (**G**) lateral view. Rostrum of *Velociraptor* in (**H**) lateral and (**I**) ventral views. Rostrum of *Gorgosaurus* (cast) in (**J**) lateral view nar, naris; pmf, premaxillary foramina; max, maxilla; pmd, premaxillary dentition; md, maxillary dentition. Scale bar = 9 mm (**A,B**), 100 mm (**C,D**), 50 mm (**E**–**F**), 10 mm (**G**), 20 mm (**H**–**I**).

**Figure 2:**
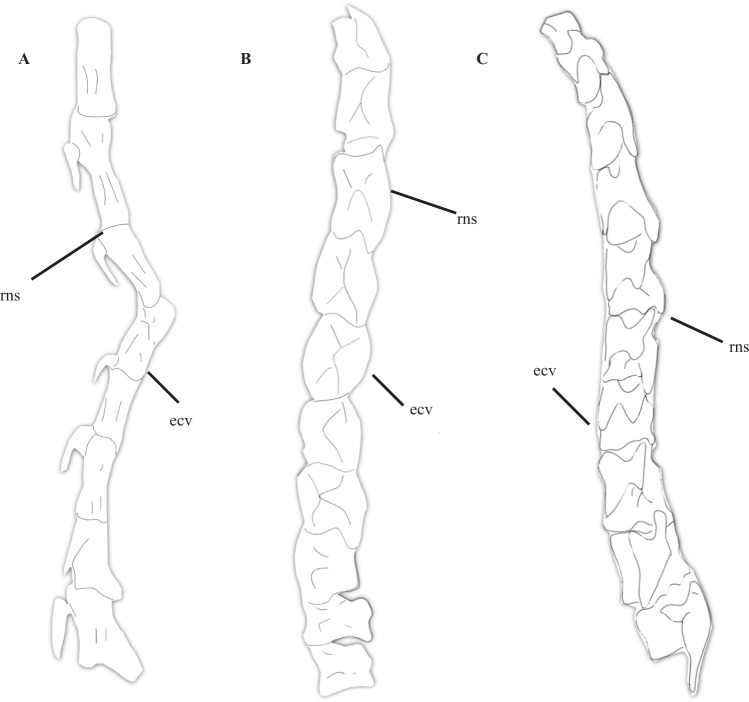
Comparative anatomy of the cervical series in selected theropods. Cervical series of *Halszkaraptor* (**A**) after Cau *et al*.^32^, *Falcarius* (**B**) after Zanno^3^, and *Struthiomimus* (**C**) after Osborn^104^.

**Figure 3:**
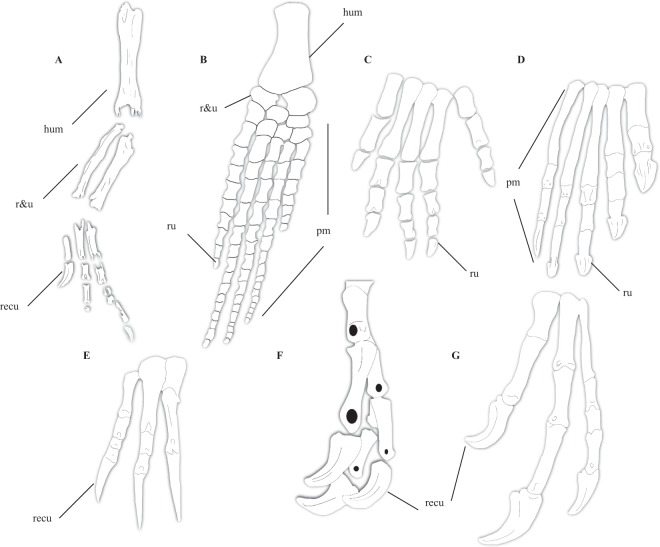
Comparative anatomy of the forearm of *Halszkaraptor* and selected tetrapods. Forelimb of *Halszkaraptor* (**A**), forelimb of *Muraenosaurus* (**B**) after Andrews^105^, manus of *Tanystropheus* (**C**) after Nosotti^88^, manus of *Araripemys* (**D**) after Meylan^89^, generalized manus of a therizinosauroid (**E**) after^2,3^, and (**F**) manus of *Deinocheirus* (pers. obs. of *Deinocheirus* cast at AMNH). hum, humerus; r&u, radius and ulna; ru, rounded unguals/ultimate phalanges; pm, paddle-like morphology; recu, recurved unguals.

One of the features used to support an aquatic lifestyle for *Halszkaraptor* is the presence of an extensive system of neurovascular canals in the premaxillae of this taxon (Fig. 1A–J)^32^. This characteristic is present in a variety of semiaquatic and aquatic piscavorous tetrapods, including crocodylians^33^. As Cau *et al*.^32^ noted, extensive neurovascular systems are also found in a variety of terrestrial theropods^34–36^. Although *Halszkaraptor* was differentiated from other theropods in possessing a rostral neurovascular system not entirely restricted the lateral portions of the premaxillae^32^, the rostral neurovasculature extends onto the dorsal surface of the body of the premaxilla in basal members of most other maniraptoran clades. In the basal therizinosaur *Jianchangosaurus*, the premaxillae are covered with neurovascular foramina that are present on both the lateral surface of the premaxillae and the medial portion of the dorsal surface (including the subnarial fossa) of each bone (see Fig. 3b in 18, Fig. 1E)^18^. In the more derived therizinosaur *Erlikosaurus*, the same morphology, where the premaxillae harbor neurovascular foramina on both their lateral and mediodorsal surfaces, is clearly present (see Lautenschlager *et al*.^19^ for clear scans of the premaxillae of *Erlikosaurus*; Fig. 1C,D). Basal ornithomimosaurs like *Shenzhousaurus* also show neurovascular foramina on the lateral, anterior, and subnarial surfaces of their premaxillae^13^ (Fig. 1F; although note the possible absence of any clear neurovasculature in the anterior skull of *Hexing*)^37^, which, as in therizinosaurs for which the premaxillae are known (e.g., *Jianchangosaurus*, *Erlikosaurus*), were laterally expanded^12,13,37^. In the derived deinocheirid ornithomimosaur *Deinocheirus*^17^, numerous neurovascular foramina are present towards on the mediodorsal, anterior, and lateral surfaces of the premaxillae (Fig. 2 in 41). In the related *Garudimimus*, a similar distribution of foramina is present^34^. This distribution of foramina on the anterior, lateral, and dorsal surfaces of the premaxillae is well-documented for ornithomimid (*Gallimimus*, *Struthiomimus*, *Ornithomimus*, *Sinornithomimus*, etc.) premaxillae and dentaries, where rhamphotheca are present and probably relate to the development of the neurovasculature^14,16,19,35,36^. In the basal-most alvarezsaur *Haplocheirus*, neurovascular foramina are present on the lateral, anterior, and mediodorsal surfaces of the body of the premaxillae, as in other alvarezsaurs and theropods^38^. The premaxillae of definite basal oviraptorosaurs where the skull is preserved bear similar distributions of foramina. In *Incisivosaurus gauthieri* and *Caudipteryx* *zoui*, numerous foramina are scattered along the lateral surface of the premaxillae, with one larger foramen placed ventral to the anterior end of the naris in the former taxon^39,40^. The skulls of both these taxa are moderately to entirely crushed, meaning that a precise understanding of the distribution of foramina on the premaxillae is not attainable^39,40^. This is also the case for the bizarre scansoriopterygids, which may be basal oviraptorosaurs^41^. In caenagnathoid oviraptorosaurs, numerous neurovascular foramina are present across the whole surface of the premaxilla^8^. As in ornithomimosaurs^5,16^, numerous neurovascular foramina are also present on the lateral, anterior, and ventroanterior surfaces of the dentary in some oviraptorosaurs^42^. Therefore, the presence of neurovascular foramina on the lateral, anterior, and dorsal portions of the exposed surface of the premaxillae is found in basal to derived members of the Ornithomimosauria, Therizinosauria, and Alvarezsauria, and intermediate and derived members of Oviraptorosauria, undermining Cau *et al*.’s claim that a neurovascular system present on the dorsal, in addition to lateral, surface of the premaxillae distinguishes *Halszkaraptor* from other maniraptorans. Because this feature is present in basal-intermediate and and derived members of all major clades of non-paravian maniraptorans and maniraptoriforms (Maniraptora + Ornithomimosauria), it is most probably plesiomorphic with respect to Maniraptora and secondarily lost within dromaeosaurids more derived than *Halszkaraptor*. Furthermore, it is important to note that the presence of extensive neurovasculature is not unique to semi-aquatic forms. The presence of extensive neurovasculature was previously used to support a semiaqautic lifestyle in spinosaurs^43,44^.

Recent research into the neurovasculature of tetanuran theropods like *Neovenator*^45^ has shown that many different predatory theropod clades possessed complex and extensive neurovasculature, and that superficial comparisons with the neurovasculature seen in groups like crocodylians is unwarranted. In coelurosaurs, extensive neurovasculature in the premaxilla, maxilla, and dentary may be related to the development of rhamphothecae^2,4,5,8,11,14–19,35,36,39,41^, which in turn is probably related to the acquisition of omnivorous or herbivorous diets^11,15–18^. Laterally, the neurovasculature of the premaxillae of *Halszkaraptor* resembles the condition in other dromaeosaurids and hypercarnivorous theropods like tyrannosauroids, where numerous foramina appear on the surfaces of the premaxillae (Fig. 1A,G–H,J)^32,45^.

### Platyrostral premaxillae

*Halszkaraptor* differs from other dromaeosaurids in possessing low, laterally expanded (“platyrostral) premaxillae that contain the extensive neurovascular system found in this taxon (Fig. 1A,B,H,I). Cau *et al*.^32^ drew comparisons between this feature in *H*. *escuilliei* and spinosaurids, the preserved gut contents of which include fish remains^44^. Cau *et al*.^32^ compared the construction of the skull of *H*. *escuilliei* to the anterior skulls of modern birds like ducks and geese, with which *Halszkaraptor* was considered somewhat analogous. However, moderately to strongly (=platyrostral) laterally expanded premaxillae are found in a variety of maniraptorans and maniraptoriforms, including primitive and intermediate ornithomimosaurs like *Shenzhousaurus*^13^ and *Harpymimus*^46^, derived ornithomimid ornithomimosaurs^14,16,19,35,36^ and deinocheirids^17,34^, where they likely acted as an anchorage point for enlarged rhamphotheca^4,5,16,17^. The morphology of the premaxillae in ornithomimosaurs has often been likened to the beaks of birds^4,5,35,36,47^ *Jianchangosaurus* and *Erlikosaurus* both show laterally divergent premaxillae that also seem to have supported rhamphotheca (Fig. 1C–E)^18,19,48^. Among these, the premaxillae of *Erlikosaurus* are the best preserved and are highly reminiscent of the premaxillae of *Halszkaraptor* in their clear lateral expansion in dorsal view (Fig. 1D)^19,48^, compared to the mediolaterally thin rostra of other dromaeosaurids (Fig. 1I)^20,21^. However, basal^7^ and derived alvarezsaurs like *Haplocheirus*^38^ and *Shuvuuia*^49^ show the mediolaterally ‘thin’ condition. In even the most basal oviraptorosaurs, the skull is very bizarre compared to other coelurosaurs^8,39,40^. However, in these and more derived oviraptorids^8,50,51^ and caenagnathids (based on the mediolaterally widened dentaries)^42,52^ the premaxillae do not show the heavily mediolaterally compressed condition present in all dromaeosaurids besides *Halszkaraptor*^20,21,32^, troodontids^53^ and alvarezsaurs^38,49^. However, the premaxillae of oviraptorosaurs are dorsally expanded to form a crest in most taxa^8,50–52^, contrasting with the condition in *Halszkaraptor*. Because this feature seems to be variously present and absent in basal and derived members of clades along and bracketing Maniraptora, it is premature to consider laterally expanded premaxillae a plesiomorphic state for maniraptoriforms. Nonetheless, the presence of laterally expanded to strongly platyrostral^17^ premaxillae in a variety of maniraptoriforms with diverse bauplans indicates this feature is not at all suggestive of a semiaquatic lifestyle in *Halszkaraptor*. The condition of platyrostral premaxillae in *Halszkaraptor* and some other maniraptorans is vaguely reminiscent of the premaxillae of herbivorous dinosaurs like the rebbachisaurid Nigersaurus^53^, titanosaurs^54–56^, and hadrosaurids^57^. Given this evidence, platyrostral premaxillae in theropod, sauropod, and ornithopod dinosaurs is probably related to omnivory or herbivory, which was probably present in most—if not all—of the maniraptoran clades bracketing Paraves^5,11,15–17^.

### Retracted, elongate nares

It is unclear how Cau *et al*.^32^ observed retracted nares in *Halszkaraptor*, as the anterior nasals are not preserved in that taxon. Their reconstruction of the skull of *H*. *escuilliei* restores the nares as elongate fenestrae extending posteriorly into the first fifth of the nasals. However, the anterior margins of the nares on the premaxillae are comparable in curvature to those of other dromaeosaurids^20,21^, where the ratio between the semi-major and semi-minor axes is smaller than in the restoration of the complete skull by Cau *et al*.^32^ (Fig. 1A,G,H). In this way, Cau *et al*.’s^32^ reconstruction of this portion of the skull in *Halszkaraptor* is slightly inaccurate, given that the preserved portion of each naris in this taxon does not differ extensively from the corresponding portion in other dromaeosaurids. Slightly retracted nares are also present in some other paravians, including the troodontid *Mei long*^58^. In the therizinosaurs *Erlikosaurus* and *Jianchangosaurus*, elongate nares extend posteriorly above a third or more of the maxillae (Fig. 1C–E)^18,19^. Elongate nares extending over at least a third of the maxillae are also clearly present in the basal oviraptorosaurs *Incisivosaurus*^39^ and *Caudipteryx*^40^, the giant ornithomimosaur *Deinocheirus*^17^, and non-maniraptoriform coelurosaurs, like the basal tyrannosaurs *Guanlong*^59^ and *Proceratosaurus*^60^, and the possibly coelurosaurian megaraptorans^61^. More derived tyrannosaurs also show elongate nares, although not to the extent seen in more basal taxa (Fig. 1J). Basal troodontids like *Mei long* and the recently described *Liaoningvenator curriei* also show this elongate condition of the nares^58,62,63^ Cau *et al*.^32^ also rightly note in the supplementary text of their paper that several early avialians also show nares somewhat similar to that of *Halszkaraptor*. Given the presence of this characteristic in basal members of the majority of major coelurosaurian clades (Tyrannosauroidea, Therizinosauria, Oviraptorosauria, Troodontidae, Aves), it may be that elongate nares are plesiomorphic with respect to Coelurosauria^60,61^, with the absence of elongate nares in basal ornithomimosaurs explainable due to the extreme elongation of the maxillae in the most basal members of that clade^12,13^. Basal alvarezsaurs also display moderate elongation of the maxillae, which may also explain the absence of elongate nares in their skulls^7,38^. Notably, the bizarre possible maniraptoran *Fukuivenator* also displays an enlarged naris that might have extended over a third of the maxilla, although this feature remains to be verified^64^. Despite the support for it found here, if the presence of elongate nares is not found as the plesiomorphic state for coelurosaurs in future analyses, the presence of them in a variety of theropods that do not show any features for a semiaqautic lifestyle provides evidence against the argument of Cau *et al*.^32^, who argued this feature was indicative of such an ecology. The ‘dorsally-oriented’ nature of the nares of *Halszkaraptor* as described by Cau *et al*.^32^ also does not substantially differ from the condition in some other coelurosaurs, such as therizinosaurs^18,19^. Furthermore, the comparison drawn between *Halszkaraptor* and spinosaurs by Cau *et al*. is misleading, as in spinosaurines the naris is relatively small and sits closer to the center or posterior end than the anterior end of the skull^44,65^. Rather, the condition in *Halszkaraptor* is more closely comparable to that in baryonychine spinosaurs like *Suchomimus*^66^ and *Baryonyx*^43^, which do not seem to have been adapted for an aquatic lifestyle like some spinosaurines^44^. The anterior portion of the skull of *Halszkaraptor* is clearly not as flattened as those of crocodylians, such as alligators^33^. Therefore, there is little evidence to suggest the nares of *Halszkaraptor* were different from those of basal members of other coelurosaurian clades in a way that might indicate a novel ecology for this taxon.

### Dentition

As in some herbivorous maniraptorans and paravians, the teeth of *Halszkaraptor* lack serrations entirely^15,21^. As in almost all other other dromaeosaurids, many troodontids, avians, tyrannosaurs, basal oviraptorosaurs, and *Fukuivenator*^20,21,39,40,53,58,62–64^, the dentition of *Halszkaraptor* is slightly heterodont, with the premaxillary and maxillary teeth showing slightly different morphologies as in other dromaeosaurids^20,21,32^. One interesting feature of the premaxillary teeth of *Halszkaraptor* described by Cau *et al*.^32^ was their delayed replacement rate. A large amount of research into the loss of teeth in some maniraptoran dinosaurs has found a delayed replacement rate to be linked to tooth loss in several clades, including therizinosaurs and ornithomimosaurs^11,15^. As in basal members of the Ornithomimosauria like *Nqwebasaurus*^67^ and *Pelecanimimus*^12^, *Halszkaraptor* possesses a large number of premaxillary teeth^32^ On the whole, the skull of *Halszkaraptor* also shares many similarities with basal troodontids, including an increased number of maxillary teeth, tightly packed teeth, and recurved, ziphodont, unserrated crowns^15,53,58,62,63^. Therizinosaurs, such as *Erlikosaurus* and the basal taxa *Jianchangosaurus*, *Falcarius*, and *Beipiaosaurus*, also possesses an increased number of maxillary teeth (Fig. 1C,E)^3,18,19,67^, as do basal alvarezsaurs like *Haplocheirus*^7,38^, basal ornithomimosaurs like *Pelecanimimus*^12^, and the basal tyrannosaur *Proceratosaurus*^60^. Members of basal clades in the Dromaeosauridae, including microraptorans and unenlagiines, also possess a large number (20+) of teeth in their maxillae^20,21,26–30^. Basal oviraptorosaurs seem to represent the exception, possessing very few crowns^39,40^. In the phylogenetic analysis conducted, the presence of serrations on teeth is coded for by character 81, whereas the number of maxillary teeth was coded for using character 82^41,68^. Cau *et al*.^32^ drew comparisons the dentition of *Halszkaraptor* and that of marine reptiles like plesiosaurs and possibly ichthyophagous dinosaurs like spinosaurs based on features like the unserrated nature of the crowns, the large number of crowns in both the premaxilla and maxilla, and the delayed replacement rate of the premaxillary crowns. However, as I note and as Cau *et al*.^32^ noted, unserrated tooth crowns are distributed in a variety of paravians, including basal members of Aves^69^, Troodontidae^58,62,63,69^, and even Dromaeosauridae^21–23,26,29^. As I have noted, a the presence of 20 or more maxillary teeth is widespread among the basal members of almost all clades of maniraptorans and maniraptoriforms, and is also present in basal members of some non-maniraptoran coelurosaur clades. Therefore, it is likely that unserrated teeth are plesiomorphic with respect to Paraves, whereas a large number of maxillary teeth are plesiomorphic with respect to Maniraptoriformes, and derived eudromaeosaurian dromaeosaurids like *Velociraptor* simply regained serrations on their teeth and reduced their number of maxillary crowns^21^. Given that unserrated teeth are found in virtually all toothed paravians besides eudromaeosaurian dromaeosaurids and derived troodontids and a large number of maxillary teeth are found in all tooth maniraptorans besides eudromaeosaurs, there is absolutely no evidence that the presence of these features in the teeth of *Halszkaraptor* are anything but plesiomorphic features, much less adaptations to ichthyophagy as hypothesized by Cau *et al*.^32^ Furthermore, a delayed replacement rate in the premaxillary crowns of *Halszkaraptor* is shared with a variety of more basal maniraptoran taxa, suggesting this feature might be plesiomorphic as well and diminishing the apparent similarity between the teeth of *H*. *escuilliei* and marine reptiles like plesiosaurs remarked upon by Cau *et al*.^32^. However, further study of tooth replacement in maniraptorans will have to be performed before delayed tooth replacement is able to be tested for being a synapomorphy of Maniraptora or a more inclusive clade.

### Number of cervicals and elongation of cervical vertebrae

Cau *et al*.^32^ noted the comparatively long neck of *Halszkaraptor*, which, unlike other paravians, composes at least 50% of the snout-to-sacrum length in this taxon. However, given its basal position in Paraves in their combinable components topology of Coelurosauria^32^, it is unclear why Cau *et al*. allied this feature to elongate necks in derived semiaquatic avians (e.g., *Cygnus*) rather than the many long-necked (approximately 50% of snout-sacrum length) non-paravian maniraptorans and coelurosaurs. Despite the fact that Cau *et al*.^32^ claimed the neck of *Halszkaraptor* composed the greatest percentage of snout-to-sacrum length among non-avian coelurosaurs, a large number of clades include taxa that approach, reach, or possibly even exceed that threshold. These include ornithomimosaurs^4,12–14,17,34–37^, therizinosaurs^2,3,11,18^ and oviraptorosaurs (Fig. 2)^8,40,50–52^. The possible maniraptoran theropod *Fukuivenator* possessed a notably elongate neck with up to 11 cervical vertebrae^64^, one more than in *Halszkaraptor*. However, *Fukuivenator* seems to have possessed a longer caudal series than *Halszkaraptor*^32,64^. The basal therizinosaur *Jianchangosaurus* possessed 10 cervical vertebrae that produced a moderately elongate neck approximately the length of the thorax of this taxon^18^. *Beipiaosaurus* possessed 9 elongate cervical vertebrae^70,71^ and a thoracic morphology similar to other therizinosaurs, suggesting the neck made up approximately 50% of the length from the snout to the sacrum of this taxon. The precise number of cervical vertebrae in *Falcarius* cannot be determined, but the cervicals of this taxon were elongate^3^, suggesting the neck of *Falcarius* composed a similar percentage of the snout-to-sacrum length seen in other basal ornithomimosaurs (Fig. 2B, but see the reconstruction in Kirkland *et al*.^72^). More derived therizinosaurids possessed highly elongate, sometimes massively built necks that easily composed more than 50% of the length between the tip of the premaxillae and the sacrum^2,11,70,71^. The basal alvarezsaur *Haplocheirus* possessed 10 relatively elongate cervical vertebrae that form 40+% of the snout to sacrum length^73^, and the slightly more derived *Bannykus* and *Xiyunykus* seem to have possessed similar counts^7^. Among basal oviraptorosaurs, *Caudipteryx* preserves an elongate neck consisting of 12 cervical vertebrae that form approximately half of its pre-caudal length^40^. Among oviraptorids, the cervical count varies between 9 and up to 13 cervicals, with 22 to 23 presacral vertebrae usually present (closely comparable to the 22 known for *Halszkaraptor*)^8^. Oviraptorosaurs possessed a deep, shortened thorax, and the necks of some even surpassed 50% of the snout to sacrum length (see *Corythoraptor* for an extreme example)^8,50–52,73^. The cervical vertebrae of basal ornithomimosaurs, such as *Hexing* and *Pelecanimimus*, are elongate, as in more derived forms^4,12,14,35–37^. In ornithomimosaurs, there are 10 elongate cervical vertebrae (Fig. 2C) and 13 dorsal vertebrae, for a total of 23 presacral vertebrae^4^. The well-preserved nature of many ornithomimosaur specimens shows the cervical series clearly formed at least 50% of the snout-to-sacrum length in these taxa^4,12,14,35–37^. Therefore, the cervical—indeed presacral—count in *Halszkaraptor* is closely similar to that found in basal members of every single non-paravian maniraptoriform clade, and, as in all of these taxa, the cervical series is elongate and forms a large percentage (40+% of the snout-to-sacrum length).

The length of the neck of *Halszkaraptor* and the elongate nature of the cervical vertebrae in that taxon are what would be expected for the basal-most paravian and simply represent the probably plesiomorphic condition of a neck formed by elongate cervicals. Furthermore, among dromaeosaurids, the cervical and presacral counts of *Halszkaraptor* (11, 22) are only slightly greater or less than that of *Velociraptor* (9, 21)^20^, *Buitreraptor* (~10+, ~22–23)^31^ or *Linheraptor* (10,?)^74^. Furthermore, the cervicals in some of these taxa are also rather elongate and comparable to *Halszkaraptor*^31,32^. Therefore, neither the cervical count of *Halszkaraptor* are especially aberrant among dromaeosaurids, and certainly not among basal and derived members of clades bracketing Paraves. Based on the available evidence, it seems most reasonable to conclude elongate cervical vertebrae are plesiomorphic among maniraptorans, and derived dromaeosaurids like *Velociraptor* show a secondary reduction of this feature. In any case, the presence of elongate cervicals in many other maniraptorans strongly suggests the elongate neck of *Halszkaraptor* is not unique nor a clear indicator of a switch towards an ichthyophagous, semi-aquatic lifestyle. Comparisons with long-necked marine groups like plesiosaurs are therefore entirely unjustified.

### Modifications to cervical vertebrae

Several features of the cervical vertebrae of *Halszkaraptor* may be comparable to characteristics of the cervicals of some chelonians and semiaquatic birds. These are (1) neural spines reduced and ridge-like, (2) neural arches elongate, (3) postzygapophyses that are merged together, (4) cervical ribs and vertebrae fused together, and (5) zygapophyseal facets positioned horizontally. Rather heavily reduced to nearly absent neural spines are probably a plesiomorphic character state among maniraptorans, because basal and derived oviraptorosaurs^8,51–53^ basal (*Falcarius*, *Jianchangosaurus*)(Fig. 1B)^3,18^ and derived^2,3,11^ therizinosaurs, various troodontids (including *Mei long*)^53,62^ and basal and derived ornithomimosaurs (Fig. 1C)(*Hexing*, *Nqwebasaurus*, *Struthiomimus*, *Ornithomimus*, *Archaeornithomimus*, etc.)^4,17,35–37,75^. That the neural spines of the cervicals of a very basal paravian like *Halszkaraptor* are reduced is therefore unsurprising. Elongate neural arches are also regularly found in basal and derived ornithomimosaurs (Fig. 1C)^37^, and are present in basal therizinosaurs (e.g., *Falcarius*)^3^. Notably, neural spines are absent in the cervical vertebrae of the unenlagiine *Austroraptor*^27^. The complete connection of the postzygapophyses by bone surface is present in the basal-most ornithomimosaur *Nqwebasaurus*^75^ and the basal-most therizinosaur *Falcarius*^3^, and is present to a lesser extent in basal alvarezsaurs like *Aorun* and *Haplocheirus*^7,76^, the basal ornithomimosaur *Pelecanimimus*^76^, and the basal tyrannosauroid *Guanlong*^76^. The fusion of the cervical ribs to the cervical vertebrae is a feature that is also widely distributed among maniraptorans, including basal and derived therizinosaurs^2,3^, adults of some troodontids^53^, and basal^37^ ornithomimosaurs. Given that most of the features on the cervical vertebrae of *Halszkaraptor* that Cau *et al*.^32^ likened to adaptations in some aquatic tetrapods are in fact present on various other maniraptorans, maniraptoriforms, and coelurosaurs (including the possibly plesiomorphic feature of reduced neural spines), the presence of these features on *Halszkaraptor* is not especially aberrant and provides no unambiguous evidence for a semiaquatic ecology.

### Flattened forelimb bones and long bone cross-sectional anatomy

One of the main arguments given by Cau *et al*.^32^ to support the hypothesis that *Halszkaraptor* was biomechanically allied with semi-aquatic tetrapods relied on the somewhat strange anatomy of the forelimb of this dromaeosaurid. Cau *et al*.^32^ presented cross-sections of the long bones of the forelimb (the humerus, radius and ulna) and suggested the morphology of these cross sections was allied with the flattened state seen in the forelimbs of marine reptiles and diving birds^32^. However, this comparison is not precise or well-justified, as the distal humerus, radius and ulna of *Halszkaraptor* are clearly ellipsoid in cross-section (Fig. 1e–h in^32^) and clearly similar to the ellipsoid cross-sections of the upper forelimb bones of other paravians, such as *Archaeopteryx*^77^ and *Deinonychus*^78^. In contrast, the cross-sections of the forelimb bones of long-necked marine reptiles, including nothosaurs^79^ and plesiosaurs^80^, are far more flattened and do not show a clearly elliptical cross-section (Fig. 3 in 79; Fig. 2a in 80). In penguins, the cross-section of the humerus is elliptical, but far more flattened than the cross-section of the humerus of *Halszkaraptor* (compared Fig. 3 in^81^ with Fig. 1e in 32)^81^.

The morphology of the cross-sections of the long bones of *Halszkaraptor* presented by Cau *et al*.^32^ also show an additional flaw in the hypothesis that this taxon was semiaqautic. The bones of *Halszkaraptor* are clearly internally hollow to a similar extent as other paravian dinosaurs^77,78^.

However, in tetrapods adapted for a semi-aquatic or entirely aquatic lifestyle, such as marine reptiles like plesiosaurs, marine mammals, marine birds, and even spinosaurid dinosaurs, pachyostosis, the extreme thickening of cortical bone, occurs in the limbs^79–84^. Given that pachyostosis is present in the limb bones of both avian and non-avian theropods that took to the water^84^, the absence of such thickening in *Halszkaraptor*, which Cau *et al*.^32^ posit was well-adapted for a semi-aquatic ecology, would be very surprising from a biomechanical standpoint.

The absence of this feature, then, is rather telling that this taxon was probably not biomechanically suited to live in water, as its skeleton, like other paravians, would have probably been too light to keep the animal submerged. Therefore, the cross-sectional limb morphology of *Halszkaraptor* provides among the strongest evidence against a partially marine ecology in *H*. *escuilliei*.

### Modified forelimb and elongate third finger

Morphometric analyses performed by Cau *et al*.^32^ on the manual digits of select tetrapods purportedly further evinced the morphological aberrancy of *Halszkaraptor*, which plotted within the convex hull formed by “long-necked marine reptiles” (plesiosaurs, some pliosaurs, some chelonians, nothosaurs, pistosaurs) in an analysis of the ratios of digits I–III and in the hull formed by wing propelled diving birds in a principle components analysis of several features of the forelimb. These results were used to support a semiaquatic ecological mode in the taxon, with the forelimb acting as a propulsion device. However, the inferences made by Cau *et al*.^32^ from the morphometric analyses are flawed, as the forelimb of *Halszkaraptor* looks strikingly unlike the paddles formed by the forelimb bones of plesiosaurs (Fig. 3A,B). In *Halszkaraptor*, there are three distinct manual digits tipped by recurved unguals, as in virtually all other maniraptorans and all other dromaeosaurids^20,21^. Besides showing the condition of the third finger being the longest of the manual digits (also present in scansoriopterygids)^32^, nothing about the manus of *Halszkaraptor* is aberrant relative to other dromaeosaurids, coelurosaurs, or even tetanuran theropods (Fig. 3A,E–G). Cau *et al*.^32^ also remarked that, apart from the elongation of the third manual digit and metacarpal III being slightly more robust than metacarpal I, the morphology of the manus of *Halszkaraptor* and the related *Mahakala* are similar to other dromaeosaurids, showing a lack of fusion, no additional phalanges, and three elongate digits tipped with recurved unguals^20,21^. The radius, ulna, and humerus of *Halszkaraptor* also present elongate shafts, as in other dromaeosaurids, paravians, and coelurosaurs^20,21,32,41,53^. In contrast, the forelimbs of marine reptiles, such as mosasaurs^85^, plesiosaurs^86^, and ichthyosaurs^87^, consist of a massive number of flattened, heavily modified phalanges that form a distinctive paddle shape entirely distinct from the theropod manus (Fig. 3B). The striking morphological differences between the forelimb of *Halszkaraptor* and those of tanystropheids^88^ and chelonians like *Araripemys*^89^, both of which possess more digits and phalanges than *H*. *escuilliei* and other theropods and the latter of which includes highly modified, elongate manual phalanges that help form a paddle (Fig. 3C,D), also stand in contrast to this inference by Cau *et al*.^32^ *Halszkaraptor* lacks the ‘paddle’ in plesiosaurs, *Araripemys*^89^, and other aquatic vertebrates like ichthyosaurs, wherein the hand contains many closely appressed phalanges (Fig. 2). Furthermore, recent work has indicated that plesiosaurs possessed a distinctive, four-flipper-powered swim stroke that differed from that seen in forelimb-propelled diving birds^90^, casting doubt on the locomotory style Cau *et al*.^32^ implied *Halszkaraptor* might have possessed.

Another issue with this morphometric analysis was the number of paravians included. Cau *et al*.^32^ only included three definite dromaeosaurids besides *Halszkaraptor* (*Velociraptor*, *Deinonychus*, *Microraptor*), all of which are derived members of the Eudromaeosauria and Microraptoria and would not be expected to be exactly similar to *Halszkaraptor* in manual morphology (although see Fig. 3, which shows the manus of *Halszkaraptor* is clearly more similar to *Deinonychus* than to plesiosaurs and other aquatic reptiles). *Microraptor* was an arboreal glider^21^, and thus its manual proportions may have been modified for that purpose. *Velociraptor* and *Deinonychus*, in contrast, were terrestrial hypercarnivores, with heavily modified, enlarged unguals on their manual and pedal digits and distinct hands meant for grasping^20,21^. The absence of any troodontids or anchiornithids in the dataset of Cau *et al*.^32^ is also very strange and represents a clear under-sampling of paravians in this morphometric dataset. Similarly, the second principle components analysis of Cau *et al*.^32^ does not include any non-avian theropods besides *Halszkaraptor*, and so the data has not been adequately polarized with data points that could represent the control for what group the forelimb of *Halszkaraptor* is allied with. Therefore, Cau *et al*.’s^32^ hypothesis that the forelimb proportions of *Halszkaraptor* represent adaptations to an aquatic lifestyle are not at all supported by morphological and biomechanical data. Their resultant reconstruction of the glenoid facing laterally in *H*. *escuilliei* is therefore also unsubstantiated, and so the morphology of this bone in *Halszkaraptor* remains entirely unknown.

### Supratrochanteric process of the ilium

**Figure 4:**
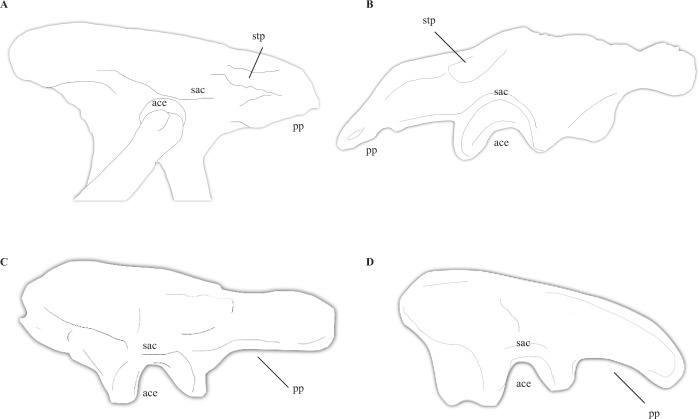
Comparative anatomy of the ilium in selected tetrapods. Ilium of *Halszkaraptor* (**A**) after Cau *et al*.^32^, ilium of *Anchiornis* (**B**) after Xu *et al*.^91^, ilium of *Tyrannosaurus* (**C**), and ilium of *Deinonychus* (**D**). ace, acetabulum; sac, supraacetabular crest; stp, supratrochanteric process; pp, posterior process.

A prominent, shelf-like supratrochanteric process was considered a synapomorphy of Halszkaraptorinae by Cau *et al*.^32^. However, this feature is widespread in maniraptoran coelurosaurs, including basal dromaeosaurids like *Unenlagia* and *Rahonavis*^21,25^, anchiornithids^91,92^, and some early avians (Fig. 4A,B)^91–93^. Cau *et al*.^32^ noted that this feature in *Halszkaraptor* had developed into a broadened shelf, as in *Mahakala* and *Buitreraptor* but not *Rahonavis*^32^. Although it is clear that the prominence of the supratrochanteric process in *Halszkaraptor* is greater than in these unenlagiines^28,32^, the supratrochanteric process in many anchiornithids is similarly developed^91,92^. Given its presence in other paravians and even other basal dromaeosaurids, this feature cannot be used to unite Halszkaraptorinae as an exclusive clade. Because this feature is present in various basal members of the three major paravian clades, Dromaeosauridae, Troodontidae, and Avialae, it is likely that the presence of a supratrochanteric process on the ilium is plesiomorphic for Paraves itself (Fig. 4A–D). Further discussion of the plesiomorphic nature of this feature can be found in the section discussing the results of the phylogenetic analysis conducted on Coelurosauria. Notably, a prominent supratrochanteric process is present in a variety of herbivorous theropods, including some therizinosauroids^2,11^ and the bizarre Jurassic herbivorous theropod *Chilesaurus*^94^.

### Shortened caudal series

*Halszkaraptor* possesses a highly modified caudal series, a feature that Cau *et al*.^32^ used to support a modified posture in this taxon analogous to some birds. However, this feature (defined here as a caudal series shorter than or equal to the snout-sacrum length) is shared with a variety of basal taxa along the maniraptoran stem, including the basal therizinosaur *Beipiaosaurus*, which possesses a pygostyle-like structure^95^, a number of derived therizinosaurs^2,11,15^, basal oviraptorosaurs like *Caudipteryx* and other caudipterygids^8,40^, many derived oviraptorosaurs^8,50–52,73^, some basal troodontids^53,63^, anchiornithids^91–93^, and early avialans^69,93^. Therefore, there is little reason to believe the posture of *Halszkaraptor* was especially aberrant in any way from many other maniraptoran and paravian dinosaurs, despite the fact that other dromaeosaurids have a more elongate tail and probably took up a different posture from *H*. *escuilliei*^20,21^. Instead, many basal members of maniraptoran clades display a shortening of the caudal series. Given that a short tail is present in many basal members of paravian and non-paravian maniraptoran clades, this feature may also be plesiomorphic with respect to maniraptorans. Further discussion of this possibility follows in the discussion.

### Metatarsus and pedal digits

**Figure 5:**
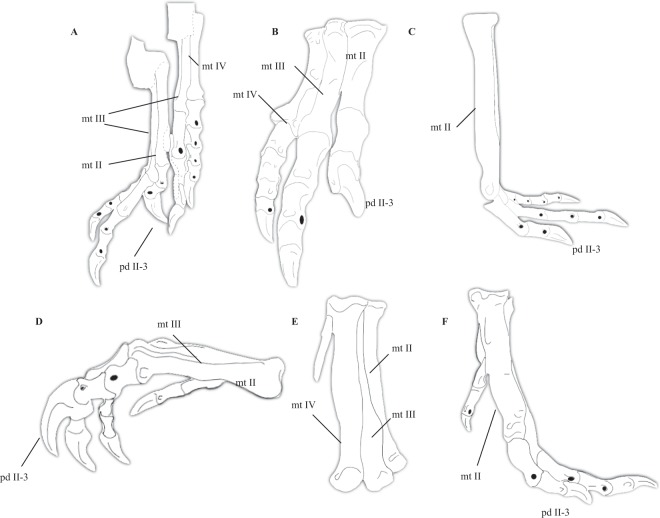
Comparative pedal anatomy of *Halszkaraptor*. (**A**) Left and right pes of *Halszkaraptor* after Cau *et al*.^32^. (**B**) Right pes of *Allosaurus*. (**C**) metatarsus of *Struthiomimus* in lateral view, (**D**) pes of *Deinonychus* in medial view. Generalized tyrannosaur metatarsus in (**E**) dorsolateral and (**F**) medial views. mt II, metatarsal II; mt III, metatarsal III; mt IV, metatarsal IV; pd II-3, pedal ungual II-3.

Most of the features shared by *Halszkaraptor* and other halszkaraptorines are in their metatarsals^32^. Among halszkaraptorines, *Mahakala* has the longest metatarsus^23^, but the metatarsals of all three members of this clade are more elongate than in more derived dromaeosaurids and lack adaptations for a cursorial lifestyle^21,32^. One notable feature is the unconstrained nature of the proximal end of metatarsal III (Fig. 5A)^32^. In many coelurosaurs, including derived tyrannosauroids, ornithomimids, deinocheirids, troodontids, and alvarezsaurs, the metatarsals are closely appressed together and interlock proximally to form a single unfused unit. In derived dromaeosaurids, the subarctometatarsalian condition, where metatarsal III is mediolaterally constrained by II and IV but still visible anteriorly, is present (Fig. 5C,D,F,G)^20,21,32^. However, the morphology of metatarsal III in *Halszkaraptor* and *Mahakala* is expected, given that the basal-most alvarezsaur *Haplocheirus*^6^, the basal-most ornithomimosaur *Nqwebasaurus*^75^, the basal-most therizinosaur *Falcarius*^3^, and the basal oviraptorosaur *Caudipteryx*^40^ all possess elongate metatarsals and a dorsally visible and convex metatarsal III. Therefore, the morphology of the metatarsus in *Halszkaraptor* and other halszkaraptorines is expected given their basal phylogenetic position and aligns with the plesiomorphic nature of this feature among maniraptorans and other theropods (Fig. 5A,B)^3,6,40,75^. Similarly, the morphology of the pedal digits of *Halszkaraptor* align with its basal phylogenetic position among dromaeosaurids. Cau *et al*.^32^ noted that the ‘sickle’ claw on pedal digit II is heavily reduced in *Halszkaraptor* compared to other dromaeosaurids (Fig. 2A,E)^20,21^. Given that basal members of other paravian clades display a reduced sickle claw^20,21,53,58,62,63,91–93^ and the presence of any hypertrophied pedal ungual on digit II seems to be a synapomorphy of paravians^20,21,53^ (absent in other maniraptorans and theropods, Fig. 2B–D,F,G), the presence of a poorly hypertrophied sickle claw in very basal dromaeosaurids like *Halszkaraptor* is expected and probably represents the transitional condition.

### Amended diagnosis of Halszkaraptorinae

The reevaluation of *Halszkaraptor* above found several features used to diagnose Halszkaraptorinae to either be dubious or to be found in many other dromaeosaurids and paravians. I therefore offer the amended diagnosis of Halszkaraptorinae: basal dromaeosaurids with the combination of: necks composing 50% of snout-to-sacrum length (possible maniraptoran plesiomorphy), proximal caudal vertebrae with horizontally oriented zygapophyses and prominent zygodiapophyseal laminae, metacarpal III shaft transversely as thick as than of metacarpal I, posterodistal surface of shaft of femur possesses an elongate fossa bounded by a crest; proximal metatarsal III unconstrained and anteriorly convex (maniraptoran plesiomorphy).

### Phylogenetic results

**Figure 6:**
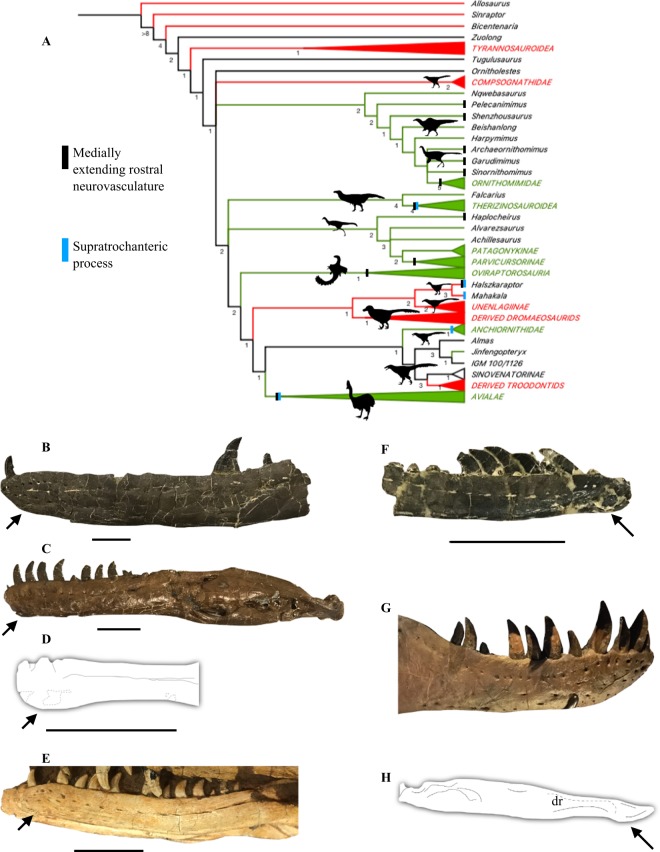
Phylogenetic relationships of *Halszkaraptor* and rostral coverings in maniraptorans. Strict consensus topology (**A**) recovered from the phylogenetic analysis of Coelurosauria. Clade diets follow Zanno and Makovicky^15^ (red = inferred carnivory; green = inferred herbivory). Tree length = 3306; Consistency index = 0.329; Retention index = 0.762. Silhouettes by the author. Dentaries of (**A**) *Deinonychus*, (**B**) *Dromaeosaurus*, (**C**) *Halszkaraptor* after Cau *et al*.^32^, (**D**) *Velociraptor*, (**E**) “*Bambiraptor*,” (**F**) *Tyrannosaurus*, and (G) *Struthiomimus* in lateral view. Arrows point to downturned ‘chins’ at the dentary symphysis. dr, lateral dentary ridge scale bar = 20 mm (**B–F**).

In light of this anatomical reassessment of *Halszkaraptor*, I reevaluated the phylogenetic position of this taxon using the matrix of Cau *et al*.^68^ and conducting a phylogenetic analysis on the modified dataset. The resulting phylogenetic analysis produced >99,999 most parsimonious topologies, each of a branch length of 3306 steps. The strict consensus topology is in Fig. 6.

I could not recover the topology found in Cau *et al*.^32^ Instead *Halszkaraptor* and *Mahakala* (the two halszkaraptorines included in the dataset) form the sister clade to Unenlagiinae, a group of peculiar paravians from the southern continents^20–31^. This clade is united by five characters: 27 (0, maxillary fenestra situated at anterior border of antorbital fossa), 107 (1, Sacral vertebrae number is six), 193 (1, ascending process of astragalus short and slender), 580 (0, sagittal crest of parietal comprised of two parallel crests), and 828 (0, Meckelian groove centered).

Nesting as a relatively basal dromaeosaurid (Fig. 4), *Halszkaraptor* would expectedly show several plesiomorphic traits. Several of the features discussed in this paper, including unserrated teeth, a large number of maxillary teeth, elongate nares that extend over 1/3 of the maxillae, medially expanded neurovasculature, a neck consisting of ~10 elongate cervicals that makes up ~50% of snout-sacrum length, reduced to nearly absent neural spines on the cervical vertebrae, the presence of a supratrochanteric process on the ilium, a shortened caudal series, a non-hypertrophied ungual on pedal digit II, and a metatarsus where metatarsal III is clearly visible and convex in dorsal view are considered here to be probable plesiomorphic states for Paraves, Maniraptora, or Maniraptoriformes in this text. The phylogenetic analysis allowed for the possible plesiomorphic nature of several of these features to be tested. The results of the phylogenetic analysis conducted provides support for the recognition of several features relevant to the body plan of *Halszkaraptor* as plesiomorphies of Paraves or larger clades. These include the posterior extent of the nares (char. 23 [1–>0] found as a synapomorphy of Dromaeosauridae, indicating nare size reduction and supporting *Halszkaraptor* as the transitional form), unserrated teeth (char. 81, found as a synapomorphy of Eudromaeosaurs [2 or 1–>0], reflecting the complete re-emergence of serrations on all teeth, and found as a synapomorphy of derived troodontids [2–>0], reflecting the partial re-emergence of denticles on some crowns; these data indicate unserrated crowns (state 2) are plesiomorphic to Paraves), a large number of maxillary teeth (char. 82, distributed throughout Coelurosauria, *Pelecanimimus may* represent an increase in Ornithomimosauria [0–>1], although the maxillary tooth count of *Nqwebasaurus* is not known), cervical vertebrae number (char. 90, found to change states [0–>1] in only therizinosaurs (increased to 12 or more cervicals), suggesting ~10 cervicals are plesiomorphic to coelurosaurs; note that the cervical count of *Falcarius* is not precisely known), caudal vertebrae number (char. 119 [0–>2], found as a synapomorphy of Maniraptora and showing reduction of caudal number from 40+ to 25–35 vertebrae), neural spine height (char. 660, shared among the coelurosaurs sampled), a non-arctometatarsalian metatarsus where metatarsal III is fully visible dorsally (char. 200, [0–>1] in derived enantiornithines, [0–>3] in derived alvarezsaurs, [1–>2] in derived ornithomimosaurs, [0–>1] in derived unenlagiines), and a non-hypertrophied ungual on pedal digit II (char. 201, [0–>1] in Dromaeosauridae). Character 826, which documents the anteroposterior length of the premaxilla compared to the maxilla (a feature clearly relevant to the discussion of the premaxilla herein), was found to be reduced [0–>1] in alvarezsaurs, suggesting the plesiomorphic condition for maniraptorans is an elongate premaxilla. Given that the premaxillae of *Halszkaraptor* form a u-shaped outline (a feature possibly relevant to their lateral expansion), I also assessed changes in character 24 (outline of premaxillae in ventral view) for Coelurosauria. In tyrannosaurs, this character changes from being v-shaped to u-shaped [0–>1], and derived tyrannosauroids have a more pronounced version of state 1 [1–>2]. Otherwise, this feature is distributed among various coelurosaurs, with little discernible pattern. Therefore, it is likely many of the features of *Halszkaraptor* simply represent the plesiomorphic states for Paraves, Maniraptora, and Coelurosauria and were secondarily changed in more derived dromaeosaurids.

## Discussion

Among dromaeosaurids and other paravians, *Halszkaraptor* possesses a clearly distinctive set of features that compose a superficially bizarre body plan^32^. While I agree with the initial assessment of *Halszkaraptor* as a relatively aberrant form among paravians, within the context of other maniraptorans and coelurosaurs, the anatomy of *H*. *escuilliei* stands out far less. Comparisons with maniraptorans in clades bracketing Paraves suggests that *Halszkaraptor* and other halszkaraptorines show many anatomical features transitional between those in non-paravian maniraptorans and mantraptoriforms and more derived, hypercarnivorous dromaeosaurids. These are found throughout the skeleton and include features on the rostrum, cervical vertebrae, manus, pelvis, pes, and caudal vertebrae. The results of phylogenetic analysis^41,68^ and the review of maniraptoran anatomy conducted above strongly posits many of these features as plesiomorphies. Additionally, a few features in *Halszkaraptor* described by Cau *et al*.^32^, particularly several in the forelimb, are not distinct from other non-avian theropods, contrasting with the initial description.

Two possible plesiomorphic features in *Halszkaraptor* not assessed in the phylogenetic analysis are the presence of medially-extending neurovasculature in the rostrum and a prominent supratrochanteric crest on the ilium. Given that anchiornithids, which are resolved as the basal-most troodontids, and early avians possess a supratrochanteric process on their ilia^92,93^, this feature is considered plesiomorphic with respect to Paraves. This feature has been considered widespread among dromaeosaurids and other paravians and therefore a possible plesiomorphic feature before^21,91,92^. There is also strong evidence to suggest that expanded neurovasculature is present in all known clades of non-paravian maniraptoran, although the condition in *Halszkaraptor* does indeed differentiate that taxon from more derived dromaeosaurids with mediolaterally thin skulls. These features are plotted on the phylogenetic tree in Fig. 6 to better show their distribution among maniraptorans and maniraptoriforms.

The slowly-replacing premaxillary teeth of *Halszkaraptor* are also reminiscent of adaptations found in herbivorous theropod lineages like therizinosaurs^11,15^, as is the lack of cursorial hindlimb adaptations in the metatarsus of *H*. *escuilliei*^2,3,11,32^. It is notable that anchiornithids were found have maxillary teeth that were highly variable in height with gaps available for replacement, a reversal of the plesiomorphic state of having isodont teeth with no replacement gaps (char. 246 [1–>0]). However, the lack of information on the presence of the former feature in various basal maniraptorans means that an assessment of whether the feature is a plesiomorphy must wait.

Notably, many of the features that ally *Halszkaraptor* with basal paravians and non-paravian maniraptorans, such as unserrated teeth, heterodonty in the teeth, a slow tooth replacement rate, a large number of teeth, an elongate neck, a shortened caudal series, and a prominent supratrochanteric shelf on the ilium, have been linked with a trend towards herbivory in ornithomimosaurs, therizinosaurs, oviraptorosaurs, *Fukuivenator*, and some troodontids^11,13–15,64^. The apparently plesiomorphic nature of several of these features, and the presence of several of them in other basal paravians, indicates *Halszkaraptor* and other basal forms, such as the anchiornithids, might have conserved portions of what constituted a body plan adapted for an omnivorous or herbivorous ecology in early maniraptorans. Although it is highly unlikely that *Halszkaraptor* was an omnivore or herbivore given the presence of many ziphodont teeth in its jaws and a sickle claw on its pes^15,32^, this taxon is important for suggesting, along with other basal paravians, that aspects of body plans not strictly adapted for carnivory were conserved during the evolution of Paraves. *Halszkaraptor* therefore documents the extensive mosaicism that occurred during this step in the development of the avian body plan. This taxon documents a point in dromaeosaurid evolution where the group was beginning to developed a heavily specialized hypercarnivorous body plan^20,21^. Along with unenlagiine dromaeosaurids, *Halszkaraptor* indicates enlarge sickle claws and tooth serrations appeared only in intermediate and derived dromaeosaurids^25–32^. The skull of *Halszkaraptor* is also more similar in shape to basal troodontids like *Mei long*^53,58,62,63^ than to the robustly-built skulls of taxa like *Dromaeosaurus*, *Deinonychus*, or *Saurornitholestes*^20,21^.

The recovery of Halszkaraptorinae and Unenlagiinae is also notable, given that members of the latter clade have occasionally been considered as specialist piscivores^28,29,32^. This hypothesis has mainly been based on both the presence of certain morphological features, including elongate skulls^21,27,28,30^ and unserrated, recurved and ridge teeth, in members of this group, as well as the recovery of their fossils from lacustrine or fluvial settings^29^. Given that the anatomy of *Halszkaraptor*, here shown to be made of a mosaic of plesiomorphic features, was originally interpreted as indicative of an aquatic ecology, a reevaluation of reported specializations for piscivory of unenlagiines is warranted. As I noted previously in this paper, unserrated teeth are plesiomorphic with respect to Paraves. Furthermore, many eudromaeosaurs have been recovered from lacustrine and fluvial settings, but piscivory has seldom been suggested in these animals^20,21^. The best evidence for dietary preferences in dromaeosaurids comes from the taxon *Microraptor*, which seems to have had a varied diet that included fish^96^, birds^97^, and lizards^98^. I therefore conclude that while *Halszkaraptor*, unenlagiines, and other dromaeosaurids probably occasionally consumed fish and other aquatic organisms, there is little unambiguous evidence to suggest they were highly specialized to do so. In *Halszkaraptor*, this assessment is additionally supported by observation of the environment represented by the formation from which the holotype was retrieved. The Djadochta Formation, from which the dromaeosaurids *Velociraptor*, *Tsaagan*, and *Mahakala* are also known^20,21^, preserves a highly arid environment that would have only harbored bodies of water in the form of scattered oases amongst sand dunes^99,100^. Such an ecosystem would have been rather inhospitable for a specialist semi-aquatic piscivore as Cau *et al*. suggested *Halszkaraptor* to be^32^. Given this environmental setting, it is hard to envision that specialized, semi-aquatic dromaeosaurs would populate this ecosystem.

Reevaluation of the premaxillae of *Halszkaraptor*, which seem more allied to non-paravian maniraptoriforms than to derived dromaeosaurids and troodontids, raises the question of whether dromaeosaurids possessed distinctive facial textures like some other maniraptorans^4,5,8,11–19,35,36^. Previous work on maniraptoriforms like ornithomimosaurs and therizinosaurs suggests that several features of the upper and lower jaws, including a maxilla with a thin ventral margin, the anterior projection of the dentary symphysis, the ventral concavity and ventral displacement of the dentary and mandible, and possibly a large number of foramina all correlate with the presence of hardened keratinous coverings (Fig. 6H)^5,15,19^. In dromaeosaurids, several of these features are possibly present. *Halszkaraptor* possess a large number of foramina on the lateral, anterior, and dorsal surfaces of its premaxillae, whereas other dromaeosaurids only posses them on the lateral and anterior surfaces (Fig. 1A,B,G,H). A large number of foramina also sit at the anterior end of the dentary of dromaeosaurids, as in other coelurosaurs (Fig. 5B,C,E,F). In many dromaeosaurids, the foramina row at the posterior end of the dentary appears as a distinctive groove (Fig. 5C–F). This is present in *Velociraptor*, *Halszkaraptor*, and many other genera^20,21,26,27,32^. This feature is reminiscent of that in some birds, and differs from the state seen in tyrannosaurs^101,102^. In many dromaeosaurids, including velociraptorines, *Halszkaraptor*, *Deinonychus*, and “*Bambiraptor*” (Fig. 5)^20,21,32^, the anterior end of the ventral surface of the dentary bulges to form a chin (Fig. 5B–F), as in some ornithomimosaurs (Fig. 6H)^4,5^. In some taxa, including *Velociraptor*, *Deinonychus*, and “*Bambiraptor*,” this feature is pronounced and contributes to the slight ventral offset of the anterior end of the dentary (Fig. 5B,E,F). However, dromaeosaurids lack maxillae with a thinning margin ventrally and a concave ventral mandible^20,21^. Thus, whether dromaeosaurids possessed a facial covering along their dentaries or facial bones (premaxillae and maxillae) remains unresolved. Although some rhamphotheca-like structure might have been anchored in the various osteological correlates in the dentary of these taxa, such correlates might just represent vestiges of the more developed condition in more basal maniraptorans, with *Halszkaraptor* showing additional such structures in its premaxillae.

## Conclusions

*Halszkaraptor*, although bizarre among paravians, possesses many features that can be traced back to more basal maniraptorans. It is therefore reinterpreted as a transitional form between non-paravian maniraptorans and more derived dromaeosaurids. A reevaluation of its anatomy and an assessment of its environment shows there is little evidence for a specialized semi-aquatic ecology in *Halszkaraptor*, as was originally hypothesized. The anatomy of the premaxillae and dentary of *Halszkaraptor* might also have some implications for the facial integument of dromaeosaurids, although no strong conclusion about the nature of such coverings can be drawn currently.

The case of *Halszkaraptor* emphasizes the importance of caution in inferring the precise ecomorphology (e.g., semiaquatic piscivore) of extinct taxa based solely on their morphology. Indeed, a multifaceted approach accounting for the phylogenetic position of extinct taxa and their anatomy as quantitatively and qualitatively compared to other, related species should be used in cases where morphology provides the only data. It is certainly possible that *Halszkaraptor* was at least partially piscivorous, as seems to be the case for spinosaurids^43,44,65,66^ and possibly the massive ornithomimosaur *Deinocheirus* (which also possessed platyrostral premaxillae)^17^. However, a systematic review of the comparative anatomy of *H*. *escuilliei* shows the purported adaptations for an aquatic lifestyle present in this dromaeosaurid^32^ are not aberrant, with many widespread among coelurosaurs. Instead, this taxon is best interpreted as a basal dromaeosaurid showing many plesiomorphic features absent in more derived members of that clade.

## Materials and Methods

### Comparative anatomy

All specimens examined here are deposited in recognized institutional collections open for scientific study. I compared the morphology of *Halszkaraptor* to other theropods using the conventional methods of comparative anatomy and based assessments on both firsthand examination of some specimens and a review of the literature on theropod osteology and phylogenetic interrelationships.

### Phylogenetic analysis

I retested the phylogenetic relationships of *Halszkaraptor escuilliei* among coelurosaurian theropods using a modified version of the dataset of Brusatte *et al*.^41^ (the main matrix was copied directly from Cau *et al*.^68^, and the new codings for *Halszkaraptor* and *Mahakala* were taken from Cau *et al*.^32^). No codings were modified. Following Cau *et al*.^32^, Cau *et al*.^68^, and Brusatte *et al*.^41^, the matrix was entered into the phylogenetics program TNT v. 1.5^103^. *Allosaurus* was used as the outgroup, and phylogenetically unstable taxa (‘wildcards’) were pruned *a posteriori* following previous studies^32,41,68^. Taxa pruned for the analysis presented here included *Kinnareemimus khonkaensis*, *Hesperonychus elizabethae*, and *Pyroraptor olympius*. I followed the methodological protocol of Brusatte *et al*.^41^ in initially subjecting the dataset of 150 taxa to the “New Technology” search options. Sectorial search, ratchet, tree drift, and tree fuse options were used with default parameters. The minimum tree length was found in 10 replicates, which allows for the analysis to find a large number of tree islands. A subsequent search using Traditional Bisection and Reconnection (TBR) branch swapping was performed. Clade support was assessed using absolute Bremer values, and a strict consensus topology was generated. Inferred diets of particular coelurosaurian clades were plotted based on Zanno and Makovicky^15^.

### Anatomical terminology

I use the term “rostrum” to refer to the anterior skull bones, including the premaxillae, anterior half of the maxillae, and the nasals.

## Supplementary information

Supplementary Information

## References

[1] Gauthier J (1986). Saurischian monophyly and the origin of birds. Mem. Cal. Acad. Sci..

[2] Zanno LE (2010). A taxonomic and phylogenetic re-evaluation of Therizinosauria. J. Syst. Palaeo..

[3] Zanno LE (2010). Osteology of *Falcarius utahensis*: characterizing the anatomy of basal therizinosaurs. Zool. J. Linn. Soc..

[4] Makovicky, P. J., Kobayashi, Y. & Currie, P. J. Ornithomimosauria. In: Weishampel, D. B., Dodson, P. & Osmólska, H. eds *The Dinosauria* (Second Edition). Berkeley: University of California Press. pp. 137–150 (2004).

[5] Cuff AR, Rayfield EJ (2015). Retrodeformation and muscular reconstruction of ornithomimosaurian dinosaur crania. PeerJ.

[6] Choiniere JN (2010). A basal alvarezsauroid theropod from the Early Late Jurassic of Xinjiang, China. Science.

[7] Xu X (2018). Two Early Cretaceous Fossils Document Transitional Stages in Alvarezsaurian Dinosaur Evolution. Current Biology.

[8] Osmólska, H., Currie, P. J. & Barsbold, R. Oviraptorosauria. In: Weishampel, D. B., Dodson P. & Osmólska, H. eds *The Dinosauria*. Berkeley: University of California Press. pp. 165–183 (2004).

[9] Persons WS, Currie PJ, Norell MA (2013). Oviraptorosaur tail forms and functions. Acta Palaeontol. Pol..

[10] Currie PJ (1993). New caenagnathid (Dinosauria: Theropoda) specimens from the Upper Cretaceous of North America and Asia. Can. J. Earth Sci..

[11] Zanno LE (2009). A new North American therizinosaurid and the role of herbivory in ‘predatory’ dinosaur evolution. Proc. Roy. Soc. B..

[12] Perez-Moreno BP (1994). A unique multitoothed ornithomimosaur dinosaur from the Lower Cretaceous of Spain. Nature.

[13] Ji Q (2003). An Early Ostrich Dinosaur and Implications for Ornithomimosaur Phylogeny. Am. Mus. Nov..

[14] Osmólska H, Roniewicz E, Barsbold R (1972). A new dinosaur, *Gallimimus bullatus* n. gen., n. sp. (Ornithomimidae) from the Upper Cretaceous of Mongolia. Acta Palaeontol. Pol..

[15] Zanno LE, Makovicky PJ (2011). Herbivorous ecomorphology and specialization patterns in theropod dinosaur evolution. Proc. Nat. Acad. Sci. USA.

[16] Norell MA, Makovicky P, Currie PJ (2001). The beaks of ostrich dinosaurs. Nature.

[17] Lee YN (2014). Resolving the long-standing enigmas of a giant ornithomimosaur *Deinocheirus mirificus*. Nature.

[18] Pu H (2014). An Unusual Basal Therizinosaur Dinosaur with an Ornithischian Dental Arrangement from Northeastern China. PLoS ONE.

[19] Lautenschlager S, Witmer LM, Altangerel P, Zanno LE, Rayfield EJ (2014). Cranial anatomy of *Erlikosaurus andrewsi* (Dinosauria: Therizinosauria): new insights based on digital reconstruction. J. Vert. Paleo..

[20] Norell, M. A. & Makovicky, P. J. Dromaeosauridae. In: Weishampel, D. B., Dodson, P. & Osmólska, H., eds *The Dinosauria*. Berkeley: University of California Press. pp. 196–209 (2004).

[21] Turner AH, Makovicky PJ, Norell MA (2012). A review of dromaeosaurid systematics and paravian phylogeny. Am. Mus. Nat. Hist. Bull..

[22] Ostrom JH (1969). Osteology of *Deinonychus antirrhopus*, an unusual theropod dinosaur from the Lower Cretaceous of Montana. Peabody Mus. Nat. Hist. Bull..

[23] Turner AH, Pol D, Norell MA (2011). Anatomy of *Mahakala omnogovae* (Theropoda: Dromaeosauridae), Tögrögiin Shiree, Mongolia. Am. Mus. Nov..

[24] Norell MA (2006). A new dromaeosaurid theropod from Ukhaa Tolgod (Ömnögov, Mongolia). Am. Mus. Nov..

[25] Novas FE, Puerta PF (1997). New evidence concerning avian origins from the Late Cretaceous of Patagonia. Nature.

[26] Makovicky PJ (2005). The earliest dromaeosaurid theropod from South America. Nature.

[27] Novas FE (2009). A bizarre Cretaceous theropod dinosaur from Patagonia and the evolution of Gondwanan dromaeosaurids. Proc. Roy. Soc. B..

[28] Gianechini FA, Apesteguia S (2011). Unenlagiinae revisited: dromaeosaurid theropods from South America. Anais da Academia Brasileira de Ciencias.

[29] Gianechini FA, Makovicky PJ, Apesteguía S (2011). The teeth of the unenlagiine theropod Buitreraptor from the Cretaceous of Patagonia, Argentina, and the unusual dentition of the Gondwanan dromaeosaurids. Acta Palaeontol. Pol..

[30] Gianechini FA, Makovicky PJ, Apesteguía S (2017). The cranial osteology of *Buitreraptor gonzalezorum* Makovicky, Apesteguía, and Agnolín, 2005 (Theropoda, Dromaeosauridae), from the Late Cretaceous of Patagonia, Argentina. J. Vert. Paleo..

[31] Gianechini FA, Makovicky PJ, Apesteguía S, Cerda I (2018). Postcranial skeletal anatomy of the holotype and referred specimens of *Buitreraptor gonzalezorum* Makovicky, Apesteguía and Agnolín 2005 (Theropoda, Dromaeosauridae), from the Late Cretaceous of Patagonia. PeerJ.

[32] Cau A (2017). Synchrotron scanning reveals amphibious ecomorphology in a new clade of bird-like dinosaurs. Nature.

[33] Leitch DB, Catania KC (2012). Structure, innervation and response properties of integumentary sensory organs in crocodylians. J. Exp. Biol..

[34] Kobayashi Y, Barsbold R (2005). Reexamination of a primitive ornithomimosaur, *Garudimimus brevipes* Barsbold, 1981 (Dinosauria:Theropoda), from the Late Cretaceous of Mongolia. Can. J. Earth Sci..

[35] Russell DA (1972). Ostrich dinosaurs from the Late Cretaceous of western Canada. Can. J. Earth Sci..

[36] Kobayashi Y, Lü JC (2003). A new ornithomimid dinosaur with gregarious habits from the Late Cretaceous of China. Acta Palaeontol. Pol..

[37] Jin, L., Chen, J. & Godefroit, P. A new basal ornithomimosaur (Dinosauria: Theropoda) from the Early Cretaceous Yixian formation, Northeast China. In, Godefroit, P. ed. *Bernissart Dinosaurs and Early Cretaceous Terrestrial Ecosystems*. Bloomington: Indiana University Press. 467–487 (2012).

[38] Choiniere JN, Clark JM, Norell M, Xu X (2014). Cranial osteology of *Haplocheirus sollers* Choiniere *et al*. 2010 (Theropoda, Alvarezsauroidea). Am. Mus. Nov..

[39] Balanoff, A. M., Xu, X., Kobayashi, Y., Matsufune, Y. & Norell, M. Cranial Osteology of the Theropod Dinosaur *Incisivosaurus gauthieri* (Theropoda: Oviraptorosauria). *Am*. *Mus*. *Nov*.**3651**, 1–35.

[40] Zhou Z, Wang X, Zhang F, Xu X (2000). Important features of *Caudipteryx* - Evidence from two nearly complete new specimens. Vertebrata PalAsiatica.

[41] Brusatte Stephen L., Lloyd Graeme T., Wang Steve C., Norell Mark A. (2014). Gradual Assembly of Avian Body Plan Culminated in Rapid Rates of Evolution across the Dinosaur-Bird Transition. Current Biology.

[42] Wang S, Zhang Q, Yang R (2018). Reevaluation of the dentary structures of caenagnathid oviraptorosaurs (Dinosauria, Theropoda). Sci. Rep..

[43] Charig AJ, Milner AC (1997). *Baryonyx walkeri*, a fish-eating dinosaur from the Wealden of Surrey. Nat. Hist. Mus. Lond. Bull..

[44] Ibrahim N (2014). Semiaquatic adaptations in a giant predatory dinosaur. Science.

[45] Barker CT (2017). Complex neuroanatomy in the rostrum of the Isle of Wight theropod *Neovenator salerii*. Sci. Rep..

[46] Kobayashi, Y. & Barsbold, R. Anatomy of *Harpymimus okladnikovi* Barsbold and Perle 1984 (Dinosauria; Theropoda) of Mongolia. In Carpenter, K. ed: *The Carnivorous Dinosaurs*. Indiana University Press. pp. 97–126 (2005).

[47] Barrett PM (2005). The diet of ostrich dinosaurs (Theropoda: Ornithomimosauria). Palaeontology.

[48] Lautenschlager S, Witmer LM, Altangerel P, Rayfield EJ (2013). Edentulism, beaks, and biomechanical innovations in the evolution of theropod dinosaurs. Proc. Natl. Acad. Sci. USA.

[49] Chiappe LM, Norell MA, Clark JM (1998). The skull of a relative of the stem-group bird *Mononykus*. Nature.

[50] Balanoff AM, Norell MA (2012). Osteology of *Khaan mckennai* (Oviraptorosauria: Theropoda). Am. Mus. Nat. Hist. Bull..

[51] Lü J, Chen R, Brusatte SL, Zhu Y, Shen C (2016). A Late Cretaceous diversification of Asian oviraptorid dinosaurs: evidence from a new species preserved in an unusual posture. Sci. Rep..

[52] Lamanna MC, Sues HD, Schachner ER, Lyson TR (2014). A New Large-Bodied Oviraptorosaurian Theropod Dinosaur from the Latest Cretaceous of Western North America. PLoS ONE.

[53] Sereno PC (2007). Structural extremes in a Cretaceous dinosaur. PLoS ONE.

[54] Martínez RDF (2016). A Basal Lithostrotian Titanosaur (Dinosauria: Sauropoda) with a Complete Skull: Implications for the Evolution and Paleobiology of Titanosauria. PLoS ONE..

[55] Nowinski A (1971). *Nemegtosaurus mongoliensis* n. gen., n. sp. (Sauropoda) from the uppermost Cretaceous of Mongolia. Palaeontol. Pol..

[56] Curry Rogers K, Forster CA (2001). The last of the dinosaur titans: a new sauropod from Madagascar. Nature.

[57] Morris WJ (1970). Hadrosaurian dinosaur bills - morphology and function. *Los Angeles County*. Museum, Contributions in Science.

[58] Xu X, Norell MA (2004). A new troodontid dinosaur from China with avian-like sleeping posture. Nature.

[59] Xu X (2006). A basal tyrannosauroid dinosaur from the Late Jurassic of China. Nature.

[60] Rauhut OWM, Milner AC, Moore-Fay S (2010). Cranial osteology and phylogenetic position of the theropod dinosaur *Proceratosaurus bradleyi* (Woodward, 1910) from the Middle Jurassic of England. Zool. J. Linn. Soc..

[61] Porfiri Juan D., Novas Fernando E., Calvo Jorge O., Agnolín Federico L., Ezcurra Martín D., Cerda Ignacio A. (2014). Juvenile specimen of Megaraptor (Dinosauria, Theropoda) sheds light about tyrannosauroid radiation. Cretaceous Research.

[62] Gao C (2012). A Second Soundly Sleeping Dragon: New Anatomical Details of the Chinese Troodontid *Mei long* with Implications for Phylogeny and Taphonomy. PLoS ONE.

[63] Shen CZ, Zhao B, Gao CL, Lu JC, Kundrát M (2018). A New Troodontid Dinosaur (*Liaoningvenator curriei* gen. et sp. nov.) from the Early Cretaceous Yixian Formation in Western Liaoning Province. Acta Geoscientica Sinica.

[64] Azuma Y (2016). A bizarre theropod from the Early Cretaceous of Japan highlighting mosaic evolution among coelurosaurians. Scientific Reports.

[65] Sales MAF, Schultz CL (2017). Spinosaur taxonomy and evolution of craniodental features: Evidence from Brazil. PLoS ONE.

[66] Sereno PC (1998). A long-snouted predatory dinosaur from Africa and the evolution of spinosaurids. Science.

[67] Liao C, Xu X (2018). Cranial osteology of *Beipiaosaurus inexpectus* (Theropoda: Therizinosauria). Vertebrata PalAsiatica.

[68] Cau A, Brougham T, Naish D (2015). The phylogenetic affinities of the bizarre Late Cretaceous Romanian theropod *Balaur bondoc* (Dinosauria, Maniraptora): dromaeosaurid or flightless bird?. PeerJ.

[69] O’Connor J (2018). The trophic habits of early birds. Palaeogeogr. Palaeoclimatol. Palaeoecol..

[70] Hedrick BP, Zanno LE, Wolfe DG, Dodson P (2015). The Slothful Claw: Osteology and Taphonomy of *Nothronychus mckinleyi* and *N*. *graffami* (Dinosauria: Theropoda) and Anatomical Considerations for Derived Therizinosaurids. PLoS ONE.

[71] Clark, J. M., Maryańska, T. & Barsbold, R. Therizinosauroidea. In: Weishampel, D.B., Dodson, P. & Osmólska, H. eds: *The Dinosauria*. Berkeley: University of California Press. pp. 151–164 (2004).

[72] Kirkland JI (2005). A primitive therizinosauroid dinosaur from the Early Cretaceous of Utah. Nature.

[73] Lü J (2017). High diversity of the Ganzhou Oviraptorid Fauna increased by a new “cassowary-like” crested species. Sci. Rep..

[74] Xu, X. *et al*. A new dromaeosaurid (Dinosauria: Theropoda) from the Upper Cretaceous Wulansuhai Formation of Inner Mongolia, China. *Zootaxa* **2403**, 1–9.

[75] Choiniere JN, Forster CA, de Klerk WJ (2012). New information on *Nqwebasaurus thwazi*, a coelurosaurian theropod from the Early Cretaceous Kirkwood Formation in South Africa. J Afr Earth Sci..

[76] Choiniere JN (2010). A juvenile specimen of a new coelurosaur (Dinosauria: Theropoda) from the Middle–Late Jurassic Shishugou Formation of Xinjiang, People’s Republic of China. J. Syst. Palaeo..

[77] Voeten DFAE (2018). Wing bone geometry reveals active flight in *Archaeopteryx*. Nat. Commun..

[78] Parsons WL, Parsons KM (2009). Further descriptions of the osteology of *Deinonychus antirrhopus* (Saurischia, Theropoda). Bulletin of the Buffalo Museum of Science.

[79] Krahl A, Klein N, Sander PM (2013). Evolutionary implications of the divergent long bone histologies of *Nothosaurus* and *Pistosaurus* (Sauropterygia, Triassic). BMC Evol. Biol..

[80] O’Keefe, F. R., Sander, P. M., Wintrich, T. & Werning, S. Ontogeny of Polycotylid Long Bone Microanatomy and Histology. *Integrative Organismal Biology* **1**(1), oby007. 10.1093/iob/oby007 PMC7671113 33791514

[81] Ksepka DT, Werning S, Sclafani M, Boles ZM (2015). Bone histology in extant and fossil penguins (Aves: Sphenisciformes). J. Anat..

[82] Ricqlès, A. & Buffrénil, V. Bone histology, heterochronies and the return of tetrapods to life in water: where are we. In Mazin, J. & Buffrénil, V. eds: *Secondary adaptation of tetrapods to life in water*. Munchen, Germany: Verlag Dr Friedrich Pfeil. pp. 289–310 (2001).

[83] Houssaye A (2009). Pachyostosis in aquatic amniotes: a review. Integr Zool..

[84] Aureliano T (2018). Semi-aquatic adaptations in a spinosaur from the Lower Cretaceous of Brazil. Cretaceous Research.

[85] Russell DA (1967). Systematics and morphology of American mosasaurs. Peabod. Mus. Nat. Hist. Bull..

[86] Watson DMS (1924). The Elasmosaurid Shoulder-girdle and Fore-limb. Proceedings of the Zoological Society of London.

[87] Sander PM (2000). Ichthyosauria: their diversity, distribution, and phylogeny. Paläontologische Zeitschrift.

[88] Nosotti S (2007). *Tanystropheus longobardicus* (Reptilia, Protorosauria): re-interpretations of the anatomy based on new specimens from the Middle Triassic of Besano (Lombardy, Northern Italy). Memorie della Società Italiana di Scienze Naturali e del Museo Civico di Storia Naturale di Milano.

[89] Meylan P (1996). Skeletal Morphology and Relationships of the Early Cretaceous Side-Necked Turtle, *Araripemys barretoi* (Testudines: Pelomedusoides: Araripemydidae) from the Santana Formation of Brazil. J. Vert. Paleo..

[90] Muscutt LE (2017). The four-flipper swimming method of plesiosaurs enabled efficient and effective locomotion. Proceedings of the Royal Society B: Biological Sciences.

[91] Xu X (2009). A new feathered maniraptoran dinosaur fossil that fills a morphological gap in avian origin. Chin. Sci. Bull..

[92] Godefroit P (2013). A Jurassic avialan dinosaur from China resolves the early phylogenetic history of birds. Nature.

[93] Godefroit P (2013). Reduced plumage and flight ability of a new Jurassic paravian theropod from China. Nat. Commun..

[94] Novas FE (2015). An enigmatic plant-eating theropod from the Late Jurassic period of Chile. Nature.

[95] Xu X, Cheng Y, Wang XL, Chang C (2003). Pygostyle-like structure from *Beipiaosaurus* (Theropoda, Therizinosauroidea) from the Lower Cretaceous Yixian Formation of Liaoning, China. Acta Geologica Sinica.

[96] Xing L (2013). Piscivory in the feathered dinosaur *Microraptor*. Evolution.

[97] O’Connor J, Zhou Z, Xu X (2011). Additional specimen of Microraptor provides unique evidence of dinosaurs preying on birds. Proc. Nat. Acad. Sci. USA.

[98] Zhou, Z. *et al*. *Microraptor* with Ingested Lizard Suggests Non-specialized Digestive Function. *Current Biology*, in press (2019). 10.1016/j.cub.2019.06.020 31303494

[99] Dingus LD (2008). The geology of Ukhaa Tolgod (Djadokhta Formation, Upper Creta-ceous, Nemegt Basin, Mongolia). Am. Mus. Nov..

[100] Fastovsky DE, Adamgarav DB, Shimoto HI, Watabe M, Weishampel DB (1997). The paleoenvironments of Tugrikin-shireh (Gobi Desert, Monglia) and aspects of the taphonomy and paleoecology of *Protoceratops* (Dinosauria: Ornithischia). Palaios.

[101] Carr TD (2017). A new tyrannosaur with evidence for anagenesis and crocodile-like facial sensory system. Sci. Rep..

[102] Sedlmayr, J. C. Anatomy, Evolution, and Functional Significance of Cephalic Vasculature in Archosauria. Unpublished PhD Dissertation, Ohio University, Athens, 1–398 (2002).

[103] Goloboff P, Catalano S (2016). TNT version 1.5, including full implementation of phylogenetic morphometrics. Cladistics.

[104] Osborn, H. F. Skeletal adaptations of *Ornitholestes*, *Struthiomimus*, *Tyrannosaurus*. *Bull*. *Am*. *Mus*. *Nat*. *Hist*. **35**(43), 733–771.

[105] Andrews, C. W. A descriptive catalogue of the marine reptiles of the Oxford Clay, Part II. British Museum (Natural History), London, England (1913).

