# Author Correction: *Halszkaraptor escuilliei* and the evolution of the paravian *bauplan*

**DOI:** 10.1038/s41598-020-60007-4

**Published:** 2020-02-19

**Authors:** Chase D. Brownstein

**Affiliations:** Research Associate, Dept. of Collections & Exhibitions, Stamford Museum and Nature Center, Stamford, USA

Correction to: *Scientific Reports* 10.1038/s41598-019-52867-2, published online 11 November 2019

In this Article, the legend of Figure 2 is incorrect,

“Comparative anatomy of the cervical series in selected theropods. Cervical series of *Halszkaraptor* (**A**) after Cau *et al*.^32^, *Falcarius* (**B**) after Zanno^3^, and *Struthiomimus* (**C**) after Osborn^104^.”

should read:

“Comparative anatomy of the cervical series in selected theropods. Cervical series of *Halszkaraptor* (**A**) after Cau *et al*.^32^, *Falcarius* (**B**) after Zanno^3^, and *Struthiomimus* (**C**) after Osborn^104^. ecv, elongate cervical vertebra; rns, reduced neural spine.”

In addition, the legend of Figure 6 is incorrect,

“Phylogenetic relationships of *Halszkaraptor* and rostral coverings in maniraptorans. Strict consensus topology (**A**) recovered from the phylogenetic analysis of Coelurosauria. Clade diets follow Zanno and Makovicky^15^ (red = inferred carnivory; green = inferred herbivory). Tree length = 3306; Consistency index = 0.329; Retention index = 0.762. Silhouettes by the author. Dentaries of (**A**) *Deinonychus*, (**B**) *Dromaeosaurus*, (**C**) *Halszkaraptor* after Cau *et al*.^32^, (**D**) *Velociraptor*, (**E**) “*Bambiraptor*,” (**F**) *Tyrannosaurus*, and (G) *Struthiomimus* in lateral view. Arrows point to downturned ‘chins’ at the dentary symphysis. dr, lateral dentary ridge scale bar = 20 mm (**B–F**).”

should read:

“Phylogenetic relationships of Halszkaraptor and rostral coverings in maniraptorans. Strict consensus topology (**A**) recovered from the phylogenetic analysis of Coelurosauria. Clade diets follow Zanno and Makovicky^15^ (red = inferred carnivory; green = inferred herbivory). Tree length = 3306; Consistency index = 0.329; Retention index = 0.762. Silhouettes by the author. Dentaries of (**B**) *Deinonychus*, (**C**) *Dromaeosaurus*, (**D**) *Halszkaraptor* after Cau *et al.*^32^, (**E**) *Velociraptor*, (**F**) “*Bambiraptor*,” (**G**) *Tyrannosaurus*, and (**H**) *Struthiomimus* in lateral view. Arrows point to downturned ‘chins’ at the dentary symphysis. dr, lateral dentary ridge scale bar = 20 mm (**B–F**).’’

