# Supplementary material for: Halszkaraptor escuilliei and the evolution of the paravian bauplan
Source: Sci Rep. 2019 Nov 11;9:16455. doi: 10.1038/s41598-019-52867-2 (PMC6848195; doi:10.1038/s41598-019-52867-2)
Supplement: Supplementary file 1 — Supplementary Information [file 41598_2019_52867_MOESM1_ESM.pdf]

Supplementary Information for: *Halszkaraptor escuilliei* and the evolution of the paravian *bauplan*

Chase D. Brownstein[1]
[1]Stamford Museum and Nature Center, 39 Scofieldtown Rd., Stamford CT.

This document includes:

1. Supplementary Phylogenetic matrix.

Supplementary Dataset.

For ease of analysis, the phylogenetic matrix of Cau et al. (2015) modified by Cau et al. (2017) is provided below as it was entered into TNT for the analysis performed in this study.

```
Allosaurus_fragilis

?10000?000000010001100100010120011101100 10??

00000000000010000000000100010000000000001010100100000001001010000000000001000000000????

000000000000000?10000000000010000100101000000100010000011000000100000000000000000000100000000000?

0?000?0011000010000001000011100021011000000000200000000?0000??0000000?00000??0000000010000[01]0?

0????????0000020?00?00000001010000?0?0010000?00000000000001000010?01000??0000000?000?

000002000000000000?00000?00?00100000000020000?000100??001100000[01]0?

0000000010000000011000000000100011000000021121001001000100?1????000000000100??10?0000?

000000000000011000?100?000?0?1000000000????000?

00010010000101000000000000000010200000000000000000000100000000000020101[01]00001000000000000000000010

0000010?

0001000001020110000000000110000000010000000000000010010001000000000000000000000000000000000000000

0000000??0000000000000000000000?00000100000000000000000?0000000

Sinraptor_dongi

?10000?000?00010001000000010120000101100 10?00?00000000000010000?00000100?10?

000000000101010010000000100101000000000001??????0??1?10?0?0??00??0??????00000000?010??

01001010000001?0?100000110000001000000000000?000?000?00?000000?0?000?

0011000010010001000001000021 01???00000000000000000?0000??0000000?00000??000000010000???00?00??

00?????2?????????????1010????????????????????????????????????1??????0??000??

00000000200000000000000000000?00?00??0???00020000?0001?0??0011000??????????
```

0001000000000000000000100000110000000210210000000?010?00?100000000000000100??01?0000?00000000000?

11000?100?010?0?100?00?001000?0010000100100001010?0?000000000000112000000?00????00000010000?

00000020101??0??1000000????????????000010010?0000?000010201101010?00011000000??1?

00000000000001001000??0???000000000100?00000000000000???????00000000??0000000000000000000000000?

0010?00000?00000001????0?0

Dromaeosaurus_albertensis

?0??0010000000000010??0???0?01??01110????1111????101?1001100??00000010011111000?

0000101001???

1??11?1?0????00??????00?0?00??1?????????1?00??011000100?

0???????????????0???0000?00000??0000??0000000?00000??

00??

00??????????????????????????????????????0????0?????????010121?000??0???00?0?????????0????0??0??

0???0??????0?0100?00?00????????????????????????0????00000100000???????0???????0001001?0?0????00??

00?????0?0?10?????????????000?1000000010????000000000001120100000000?0?

00010??0??000?

0???????????????0??0?010???????????????????????0200000???????????????????????????????????0?1?0??

110????????????????000?0????????

Deinonychus_antirrhopus

?0010????1???????1??0000?0111000111000111111100?1??00001110100000?0100111?1000?0100101000?

110001100?1121011????00110111121??????1?1101110010011100000100221110101020220?01111211201?

111100000000001000110100100000000001100000?00010?0010002110000011110110000??000001220000000000?0????

000????0???????????00000??000000000000?[01]0?0??????????????20000?000?000111000?0?0010000?

00000000000000101?0???00000??0100000?000?000000000000000000000000000?0010100000????????0?1????

00111?????????0000?????00001?10?0010001?000001010??00?0???????00??0?0??0?????00001000000??01?00?

0?00000001???????????????????????0?0?1001000?000?00?????????????????00000000001120100?0000?0?????

00000000?00001000100?????00000?1101010000100??1?0000?????????????????00000?000000000?00?100?00??

0001?1??0?000010?100000000010?010??00?0000000000000000000000000??0????000000000100??1101?000110???

200?10001?00001?00

Velociraptor_mongoliensis

?00100100100001201120000101110001110001211 1?10?

0101000011101000000010011111000101001010000110001100111210111110001101111211110101111111100100111

0000010022111011102022020111122120111 11?0000000001110110100100000000011000000000 11?

00101021100000111001100000000000011200000000000?000000?0000??0000000??0000??0000?00000000[01]0?

00??000?0000020000?000?000111000?0?001000???000000000000010100???00000??0100000?

000000000000000000000000000000?0110100000010[12]21?0001101100011100000?100000000?0100001?10?

0010001?0?0001010100000 0???0??0?01??00?0????000001000000??01?0000?0000000 1001?0?0???000??

00110100?0???01000?000?000001001000?00000000000??00?112010000???????00000??????????????01001000?

000001110101?0?0?00?0100000110?01?01000000000000000000000000?000100000?000?101010?

00001001000000000010?01002000000000000000000000000000??0000100000000010000?1?10?

0011010020001000100000100?

Balaur_bondoc

???

???1?2??1?2??0???10?????????0????111[01]11101001?1103001???2???0111?202?02?0?1?

22122??????????????????2110001001000??00??1????????????0?00??0????????????????????????1??????2?

00??00000000?[01]???0??????????????20??????0???011100 0?0?

001000???010?00?????010?00???21??0?00000020?100??0000??????00???1?000000000?00??0?00000???????

010?????????1????????

00000??

??

?0100?0?0??0?00?1101010000?00?01000?0110?01??????????????000?00??00?????10100000?00??1??????

0000100100?0000?????0?0????????????000000000000000000??000????000000210????????000?10???200?

1??????1110111

Tsaagan_mangas

?0010010010000120112000010101000 1?10001211111000101100001 00??000000100?1111000101001010000110 0??

1??????0??????????????????1?????????11?

111???00??00??00?

0000????????????2110000011?00?0000000?00001????0000000????000000?0000??0000000?00000???

000???????????????????????????20000???0?0?0??

10??0????????

0121?00???0??00011?0?????????????00?0100001?10?0010001?0?00010101000?00???0??0?01??00?0????

000001000000??0??0000?0000000 1001?000???000??001101?0?0????????????????000100100000000000000??00?

11201000000 0????000000???00?00001???????????????00?

110???000?000?????????0?10?010?????

10??????????????????02?0000000??000?1?1????110????0???000?

0????????

Bambiraptor_feinbergorum

?0010??001000012010[02]00?010111000?1100012111?1000101?00011?0?0?000?0100?1111000?010010100??

1110?1100?1??100?0?1100[01]1011??2????010101101111 0?001?100000111?211?0101?20220 2?111022100???

1110000000000100011 01?00000000000011000000000 11?0010?021100??11?110??0000?000000122000?00000?0??

00000??0000??0000000?1?000??000000000100??0??0?0?0?0?0000020000?00000?011?000?0?0?1000??

000000000?00010??????00000??0000000?000?00000000000000000000000000?00?011?10000000012 1?000110??

00011101100?100000000101000 01?10?0110001?0000010001000000???0??0??1??00?0????000001000000??01?

0000?00000001??1?0?????000??001101???????????????????0????????????????0?000??00?

1120100?????????????0000000?0000100010110000?000001110101000010?0010000011 0?01?010000000?00000?

000000000?0001?000??00001?10?0?100010?100????????????????0?000?????00??00????000000??

00001000000000100?0???10000110100200?10001????01?0?

Tianyuraptor_ostromi

??01??????????????????0????111??????????????0??1????????????????????????????????0?0?0?1010??0???

0?????????0?0?0?1??01?0112????????1??00?011110?001?100000111 2?1??0?1102223?2111102 2?0???1?

110???????????00?11????000??0???1??00?0??01???1011?0???0??????1?0???????????0?????2?000?

0???????????????????????????????????????0???????????0?00?00????00000?0?00?000?0?

0??00?1?0????10???????????????0?0?????????20?

101?????????????1????????????0000?10??000??1????????????????????????????

0???

??????????????????0??????????????????????????????0????????????????????????????01?1?1???00??0?0????

000????????????????0?????0?0???00????0????0?0???0???????0???0?????0??0??????????????????0?????0???

0??????????????????????????0010?????????0?0????????????0?0??????????

Sinornithosaurus_millenii

0001??????0???????????00???1110????1000111100?1????1??0?????????00?00100??1????010100??100????????

1??1????????0???00?1??1???????01?1?11011110?0?????0000?00?201?01?112023?2?111022?2?1???1?????????

110001?1100?0?00010001??0000???0110111100200000011110 0?00?00???000???20000?000?????0??000????

0????00000??1000??00?00?0?????0?0??0????????00?0?[12]0000???0?000100?00?0??010?0??

00??????????????????????0000??0000?0000??0?0?00?????000??0?0000?00?0???0111?11000?????????01??

10000111?1100?1000000?0101000000000?0000000?0?000000001000200???0??0??1??00?0????000000000000??00?

0000?00000001??1????0??000??001101??????????0??????????????????????????001000?010112010000???????

100?0????????????????????????????0011101????00?000??????????0?01?????????????????0?0???00000??0??

00000?000?1??0?0????10?????0?00?????0?0???0100??????0????00000????001?000?????0??000100?011?????

0110????0???000????????0?

Microraptor_zhaoianus

0??????????????????100??0??010?0?1????0?01000000?????001?

1??01?2100?01???0110?1212111?01?101111111010??1100000110121111??0112023?2?1110221201?11111??00?

0001110111110?100??000?11?000????1010111100?0?0???1?110???0?00??0?00???20??0?

000??????????????????????????0?0???????00001000?[01]0?00?0000?001000200???0?000?01???10?0???

10100?000??1?0?000?????????000???????0?00?0000000000???000?00??000000000??1211111??00?021???

011011???1?1?1100?1000000??????????????????????????????

0???

?????????????????????00???1?????????????????????00?????????????00001????????01???1?10000?000???

0000??0?01??10?????0?00000?0???00????0????0?00??0????1????0000???10??0?00???0?010???????0???0?

00??000??0?000000?000?10?000000010??????????0?1??0?????1???1??00?100?

Rahonavis_ostromi

???

0???????????????011121???1?01?011112?12????????????0??11??011??????0?01111?10111120?3?2012101?

121??21110001000?011101101?0100????0??1?????0??

1000101111???

0011000[01]0????????????????2?????????????1100??????????????????????????010100????????????????000000?

000000000100000000?00000000?001??01???0?????????0?1?11?????1????????

00??

??

?100?1??0?0??0011??????0?????0?100000????01??10???0?0????00?0???00????????????00??0?00?1????0000??

0?0???????0???010????????????000?????????0000000?000?10??00?0001????????00?0?11?0??0??????????????

00

Buitreraptor_gonzalozorum

?0010????????????????00001011?00??????????100?10??01?0?????????00??001??????????0210??100?

0010111100111?110???100011012[012]121???????0110111010?1?1??0??????11?101??12[02]23??01?1?1?

120???11?0??0??1?011?0?1110000?????0??1????????110100011001100???0??????0000??0?00?10200?00?

0???????????0???????0?000101???0?????00000000000[01]0??????????0100010?0?0?00??0?10000?0?

001100??00000100000001?100????????????????000????00???0?00??????????0?00?000?0010?00??????????????

1????0010??1100?0000???00?0?????????1?11?00?0???010?0101?0??0????00000??1100????????0000???0?0??

0??0????????????????100?????000??00?0??????????????????????0?????????????????0?000???0?1?

2?????????????????000???????0001001000??0??000001110101000???0?001??000???????01000???00??

0?????????0?0?000??????????00????0?100010?0???????01000?????0?0001000000000????000000??0??

01000?0??001???????10????11100?00010001???0?0???

Neuquenraptor_plus_Unenlagia

??

???1121111111???1????1?????????????0??1001???????????01111?101111202202011?121202011?10000??0?

010??111100000????0??11????0??110110?110????????????????????????0?????

220??0?00000?0??????????????????1?????????????1100?0?0?

0011000?00000????????????0???????????????00000?0000000000000000000?00000000?

00??

??

??11011?0?????0011???01???????00100000110?01?010000000?00?

0100???00???????????000?00?00?1????10????01???????00???010??????????????00???0????000000??000010??

0?000010?????????0??111001000?????????????0?

Austroraptor

?0000???????????????????2?1?0????????12?0001?????????????????????0?011??????????210??10?????0011?

001????11???????????????????????????????00????????0???????????????????????????????????1??????0?0?

0?0??[12]1??????????0????0????????????????11?00??01??00?????????????1[12]20??0?

0??????????????????????????????????????0??00?00?????????????????????????????????????0?0?001100???

00??

0000000??

????????00?0??????????0?10100?0000010??1???????1???????????????????????????????0??01?

01????????????????????0?10?00????????????00000?000?00?????????????????0??

0010000???????????????????000???????0001001101??????????????101???????????????????????????????

000????????00?00????????????0?????0?0???????????????????????????0?0001000?????0???????0000???????

00??????0?????01?1????1?????1?0??0001?????????

Atrociraptor_marshalli

???????????????????[02]00?0??1010????????????????????????????????00??010????????0?

000101001??

???????????????????????????????????0????0????????????????21?00??0??111?????????1???????????

0??????????????????????????????0????

0??

??00001?1????1???0?

0?000?

01???

????????????????????0?????0?00????????????????????

0???0?0?????????????

0??????0????????????????????????????000??0??

1??????????????????00???????????

Utahraptor

???????????????????[02]00?0??????????0?001???????????????????????????????????????0????0101??1????

0?1100?1??1????????0??011?????????????101?1???????????0?????????0?01????????????11?????0111?00??

0?001000100???10????????11??0???????0?????0???????????1???0???????0???10???0???

0??00000??????????????????20000?

000000???00???????0??0000000000000?0000??????0?

10??00?????000001??????????????????????????1???0??00010?

0???

??

01?????????0?0?????????????0?????0????????????????00????????????0??????????????????????????????

0?0?????????????????1??????????????????????

Adasaurus_mongoliensis

?0010?????0????2???????????????0?????0?1??11?1?0?101??001??0?????0????0?1???0??????????????01100?

11?0111?10?111[01]100??011?1?1??????1?111111??????????????1022111010102?2202?1?11221001?

111000000?000?11010010010??00??00110?0?0?0001??00100?????????11????????00?0????1??????????

0?????????00??00???000?0????????????000?0010000[01]0???????????????200?0???00??

01110???00?000000000000000000000000?002??11??0???

00???21?000??0??0001?1??????????0???0??0???????????????????????????????????0?????????1??00??????

0000010??000??0??00?0?0?00?????????0???????????0????????????????????????0??????????????????0???????

11201???100000??0?01????????????????0?

0???00?0?????????000?00????10??????????????????????????00??????0??00???????????000000??0000010000??

00010???????????01????????????????????0?

Achillobator_giganticus

??????????????????????????01?1???0000101??????0?

01100?11210???????0??011?11??????????101????????????00??10220??11010102102?011011?001?21110?0????

00?000?101????0???????1???0????001??00100021100??0??100????????

0??0000????????????????????20000?0?0?0?0??

10??00???0?00000??000???????0?0000????????

0???????????????0??????????0???????????00???????????00?001???0?0?000??

1??

??10??????????????0100??????????????????????????

100000??0101?0100000?1000??????????0??????????0??01?????1??0??0??????????????01??0?????????0??

00?????????0000000???0000100???????1???????????????1?1?????0?00?????????

Saurornitholestes_langsto

???111????????????11????0?????????????????100101?00?

11000110011121011011100?1011?1?1????????????111?????????000111221?1?1?1?2?0?????????????????11??

0????00??001101??00???00?0?1??00?0000010?001000????????1110011??????0????1220???????

0????????????????????0000???????????0????????????????????????????????0???00??

1??0?????????????????????????????????????0???

00??????????0??????00111????????00?00???????????00?01??0?0?0?000?

00??000??00?1???0?

000??????????????????????????????0??0000000????????????????????0???????????????1?

0???0??????0????????????????

0???????????????1??01?????????????0?????????00??????????????????????????11??????????????????

0???????????

Saurornithoides_mongolien

?00??1?1??1101???0?110001?1000???????????2??????????????1?010?100?0010??1????000110101?????????

1???0??1?????100??1??02012020?010??10[01]1?

1111O????????????0???10?01??????0??1?0????????????010??11000??0?1?01?0?????0?000??????0??0000????0?

1000?0??0????????????0000???0????00100?

0??00????????0?

0????000000000000000?20?0210000000000201001?10???1110?1????????????00?0100000??2??11?0?0?1?

0100010100??00???0??0?????0??0??????????????00???????????????????????????????????????0???????

1????????000????????????????0?????????????????0???????00000??????????????0????????0?

0??????????????????????????????1?010????????????00????00000????????00??00????0?0???00???????????

1??????0??000?0?????0????????????????????0?00???????0?101?0?????????1???0??????0?0?00???????

Zanabazar_junior

?00101?12?110100?001?000??100000????2022000?21??0??11100??????100?001???1????

000111010100??????????????????1?1000?1020?1??

2??????????????????????????????011????2????????????00??00???11?????????0??11000000010000O????1??

00???????00?000?????001??0?00??????????????0000??0?00????????

0[01]0??

????00010000??000??2????1??0???00000201001?11???1110?1????????????00??100000??2??11?0?0?1?

0100010100?000???0??0?????0??0??????????10??000??00?0???????????????????????000?

0001101O????????????????????000?00000????0000???000?001????????????0????00000???????????????????

0????????????????????????????????????0??????????????0?0?????????000??????????????0??????00???

0??????????????????01?00?0000??????????????????????????????????0?00????100100????????????1????000?

0????????

Xixiasaurus

???????????????????01000101110??????2?22?????????????????????0?00??001????????000201??01?

0??00?

1???0???00??????????????????????1?000000???

01?0000????00???????000000???00????????????????????????

0???0??????

0??0?0?????????????????????????????????00????110100??2?

01100?0?1?01000101000000???0??0???000??

0??010?000????????????????

00000???000??????

0??????0????0?0??????????????0????????????0100???????????????0????????????????????????1?010??????

1??????????000??????????

Byronosaurus_jaffei

?????101???101?1100110001011?0??????20220??????????1?100??????0000001??11????000211??01?0?

0???????010121????????0??02??1????

0?0??0???????21??????????0????0????1???????????11000000?1?00????0??0?000??0???000000????????????

0??????????????0?0????0????

0010???

????000?????????????????????????????000200011??0????????1????????????00??100000??2?011?0?0?1?

0100010100??00???0??0?????0??0?????????????00????????????????????????????0???????????

0??????????????????00?000000????????0??00??00?????????0???????0?0000??????????

1???000?????????????0????

000????????????????????????00?0100?????????????????????????????00?????????1?0?0?1????1??????????

000?0????????

Sinornithoides_youngi

?0??01?????????????1?000??1??0????00???2???????1??????????????00??0010???????0001110?01??????11??

001???????????0011?102121011???1?00?1?101?0???100000????21??01??02??30???11?0??001??1110?????0???

1?000110001?0??0?0?1??00?0???0???0010?0??00000????0??0??0???00???0?2000000?0????????????????????

00?????1?00??000??????????[01]0?0????????0??0?20??0?00000?0??1000???0?1000???00?0?????????0??010?

0000???000000??00??0??000???00000000000000020?0210000000????????101??????1?010100??1??

000?????????????????????????????????????

0??000??

0???0?0?00?????????

0????????????????00?110101?000?000????????????????1??????0?????000???00?0???0???0?00??0??1????

0?????100????0?0?????????0???000?000???00??10?000?????0??00???00000000010????0?1?0?0101????0????

0?????0?0???

Mei_long

?0????????0????????11010?????0?11?0020121000 01?1010??100??????00010010??1?0000?021???01?

0011011100011?2110?01???0110222111?1?????111?11100?0?1?1000001?012111?1??1200302?1110221?[01]?

0111100?0101001000??110011?0?0000???00000??000100?1??0?1?000000?001?0?0000?000001020000?

000?????????00?0??0??00??0101????????0?0000010000[01]0?0?????????0100020?00?00000?011000?0?

0000000?0000000?000?01?1?0???00000???00000?000000100000??00000000000?000021?01100000??0???20???

1010??101100?1100?10?0000?110??0??0???????????0?1?01????0?????00????????????????0??????????????

00????????????????????1???0???????0?????0?????0??0?000??????????????

0????????0?0000?????????0?????????????????????????1?1?0????????????????????????????????0???????00???00?00?

0???????0?????0?????1?0?000?1001??00?0001?0?010???0???0?0000000000?000????00???0???00?

000000100?????????01???????????0?0????0?????

Jinfengopteryx_elegans

?0???????????????????1?01???2000?????????1??00?0????1???100??????00?00??0??1??0?000?1???0???

1????????????????????????02????2??200????????110111 0??????100000?????1????????????????????2???

0??????????0??????????????????0?0??00????00???????????????01?000001???0??0?0???????0????200?0?

000?????????????????????????????0???????0?000???0?0??0?0???????????????00?0???0?0?01???

0???10?

0000???????????0??11?01??????????????000?110?????0?01??1????0?????????0?????

00?????????????????????????????0???????????????????????????????0????0????

0????????????????????????????????????0??00???????????????????????????0?????????????0????????0????

1??0????00????0??????????????????????0????????????????0?0???00??????00???1?0???0????????

0????????????????000??????????????0?0????????00?????????001?0?????????????????????000????0?????

Anchiornis_huxleyi

000100?????????????11010??11100?????0?2?000??1???11???????????000??01??1?????00020???01??0?101???

00?0??010?01???01101220??01?????011111110000??100000010?21?10011020?202?1?1122121???1?1?000??1?

01?000121100010?1000?1??00?0??????0?11?011000001???0??00?????000???030000?000??????????0??????

0????0??????0??????00000?0000[01]0?0?????????000020000?0?00??0?10000????10?0???0????????????????

10?01000??0?00?00??00?00000????????????00000?0?00??0100000000?????????00??1110?1?1?100010??000?

110??0??0?01?010?0?0?1??101?00??0??00???????????????0?????????1???000??0???2??????????1?????????

00????0????0????????????????????????????????????0?000??01?????????0???????0?0000?????????0???

000??????000??????1???00??0000100000??0?01?????????0?????0?0???00?00?0??100?00??00???11?0?

0000???????0??????????0????1000????0????0??0?0000000???00?0????????00100???0???0?01?????????

000???00?0?0?

Xiaotingia

?00?????????????????1?1???111000????0?2??????????11????????????001001??1???00??020???01??0??0???

1?0?????10?01?????1??????10??????01?01111?10???1000001011?1110?1102122?????102?12[01]??????????

10????????1?010001010000?1??0?10??00???0010?011000??1???00??0?????000??22000??

000?????????????????????0000?????????0??000?10000???0????????010002????????????11?000?0?00???0???

0?0?0????0???????????0100??1?00000???0?0000???????????????????0??0?02????10000????????00??1??????

1?1100?10??000?110??0??0?01??1????0?????0???0?????0????????????????0?????????1???

00??????????????????????????????0????0????0??????????????????????????????????????0??00???1?????????

0???????????00?????????0???????????????0011??????0???000010?000???????????????0???????0???00000?

0??100?00??00?1??1?0???????0????0??????????0?????000?????0???0??000?000?????0??????????0?100???

0?????0101????0???000???01?0?0?

Aurornis

?0010???????????????0101???11100???000?1?0???????????????????????00?001????????0?020????1??

0????????0????????0????0??0?221?00??????01?01111??0???100?0??001?011????0212202 0??

10????????????????????????0??100000?000000?1??0??0??00????111?011?0000????0???000???0000???20000?

000?????????????????????????????0??????000??0???000?0??????????00???2??0????????0?0?010????????

0?????????????????????10?0??00??0?00?00?0000000????????????????0???0??????1??010000????????0???1?

1?1??1??1000100?000?110??0??0?01??10???0?1??101?001???000???????????????0?????????10??000??0??

02???????00???????????00????0???0???0???1?????????

0??????????00???????????????????????0???01???1???00??0000100000???????????????0???????0???00??0?

0??100?00??00????1?0???0????????0??????????0?????00??????0??????0?000000??????????????

00100????????????0?????????00????00???0?

Eosinopteryx

00010????????????????1?01??????000????0?1?0?????0??1??????????????00?001????????00020????1??

0????????0??????????????0??0?231?10???????1101111??0???100000?001?1?1??1?02002?20??

10?????????????????????????00?1??0001000000?1??0??0??00????011?0???00??????00?0?00???0000???20000?

0000????????????????????????????0??????0000?0?10000?0?0?????????00???2??0????0???0?1?010????????

0?????????????????????????0??00??0?00?00?000?000?????????????????0?????00??01???000???????????0???

11??1??1??1??0??0?000?11???0??0?????????????????????0?????00????????????????0?????????10??

0????????????????????????????????0????0???0???0???1?????????

0???????????0??????????????000??????0??0011??1???0???000?100000???????????????0?????0?0???00??0?

0??100??0??0??1????0?0?0????????0??????????0????100??????0??????00000000??????????????

00100?????????0?01????0????0????00?0?0?

Troodon_formosus

???1?1112?1101000001???0?011?0??????2022000021 0?0??01100????0?10??001??????????0111010100???

111110010121111 1?1000?1020??11??????????????1?010?????000010??????????0?0?2?20?11001?0??

01111000???00010000021??01????000??1?0??010???1??0??0?1?00000?0?0???????00??????????????????????

001??0?000?????0??1?1?????????

00???

?????????????????????????????????0?????00000201001??0???1110?1?????????

0000??

0?????0000??00110????????????????????0000001000??000000??0000000???????????????????00???????0?

0??00???????????????????????0??????????????????????????????????00000?000??0????????00??0???0??0????

00??01??0000?0?1??01?01?00?0?????????????0?0?????????????????0?000?10?0?10?0?0?0?????????????0??

0????????

Sinovenator_changii

?0???0002?000011110010101?1110?011?02??2??0011???1?10100????0?000?001??????000001111 0?1?????11?

10100012110?001000110222?1?????????110111???????100?0???0?211?010112[02]0302?1110221201?

11110000??10011000111??110?000000???0000110001000110011000??1??011??0000?00000??0???00000000000?

000000?00?0??0000010100[12]00??00000?0010000?????????????????20000??00?0101?

10??00?10000001000000000000000?0?00?020?0110?

00000000020?010?10??1011000???????000000?????0000?02?011??00?1?0100010?????

0???0????????0?00????1???????????????????

0????????0???00000??00???0?????????????????????00??????????????00????0??????00111??????????????0000?

0??0?01???????1??0????0?0?0???0?0????????????0?0?1?1??0?????100?????????0????0??01?01?0?0000?

00??????0?00??0???0000?0000000001?1???0?????0??1??1?????000?0????????

EK_troodontid_IGM_100_slash_44

?????0012???0???????????????1??11??????????1??????

0??1?

00000???01?1?001???00??????0?0??1?????????

0??1?

1??

??0?????????

000???

0??

???

????????0??????????1????0????????????0000????????????????????0?1??????????????????????????????

000???

IGM_100_slash_1126

?0????002?0000?11[12]01?010??2000?011?020121000110?01010100??0??000000000?11?000000211??00?

0??????????????????0????01102?????????????????????????0??1000001???211?010?020020200010131012??

111000????0??2?001210001?00000011?00?0?020000?00100?1?00000111?01?0?00?000003??????000000?????

00??00?00?0??0??0000?00?00???0?0?????????

0??0?00???0000???0100000?00??0???0?00?

00?????00?1?000?20?02????0000001020?111??0??101?001???????0?

000??

???

???

???

IGM_100_slash_1323

?00???????0???11???1?000??20000?1??020121?00?1??0?0??????00?0?000?0????1??0000021???

0000????????????????????????011?????????????????????????????????00??1??????020?20200[12]102?

1011???????00???0????0??2???01??00??0????0?0???00?0?00100?1?000000?1?01?0?00?00?003??????

0000000???00?000?00?0?????0000?0?000??0?

00??

00?????00?00?0?00?????????1000??2???????????????????????????

01??

??

??

??

???????????????

Archaeopteryx_lithographica

100?0000??000??112010010??1110?011000012100?10?0000??100111?0?000001000002?00000200??00100?1?1???

00?0??1?0?0???00210123110000????1011111111000?1100000001121110 10?12003020121022?2?2?111100000?

0001000000030?000000000110000 0??00?00011001110000100 1?01000000???0000?030000?0000000?00110??00?

0??000?00??000???00?000000000[01]000?0????????00000?0000?00000001000000 0?0011000?000??000?

0000100000??00000?000000000000?0?00000000?00?0?[01]1000000?00???000001000 01020?00001011101?

0101100?1000000110?00000?01?01?0000?0?0100?10100?000???0000?01??00?0????00???10??000?????????

000000?0?0????????000??0011000?????0??????????000000011??????00000?000000000??00???0???0???0?

0100???????000?0000????????0?0011101?10000?000?100000??0?01?01??????0????00?0???00?00?00?100000?

000?1?1000?0?0010010??000000?0?010020?1000?000000??0?000000?0000?000?????0?000010000 1001???

0111????00?100010?000000?

Confuciusornis_sanctus

10010???????????????1?000?00??0001???0??2??0??0??00??01???????000010000?10?0001?1?????????

0??????????1021?0?2???0?2????4??111?11010??13111000?1111000000112 1?1?1??12000?2?111023?2??12??

10??11??12021101003 0?000000000110 2000??00?0101??11?10001100 1??????0?????000?

123001[01]00120?????????10??00??011200??01[12]011111000000110000100100012 0000?00?0000?0??0??

010000000?000211??0010000000001010 2101011[01]?10?0000001010000000000101010010001100 0011000?

0000000[01]000???200000110111 0?00??1100?10?0000?110?00000??1?00?0000?0?01?0?10100?00??????????1??

00?0????0000000??000??01?02???0010 0000?0????????000??00???0?????????????????????????????????????

00010???0?0??00??10?????????????0???????0001?000???0????0?00111?0?1?000?100??????0??0??1?????????

0????00?0???00?00?01?100000??0??1??0?????0?????0??00???????010???????0?????0?0000?0?0???????0??

0??????0?0000100?0?0?????01????????????1????1110111

Jeholornis_prima

10????0????????1???0???0?????001??????????????????0???????????0000?1???00?00?1?1?????????

0????????0????1?0?11???02111031??01?0???01103111000 1??110000101 1?1??0??01?0???2???1?221?10???????

01????01011010030000000 00??0??1000??00?010????1???000?1?????0???0??0??0??12300?

10010???????????????????0????00?00000??00?000000100000?0??001???0000?00000?0?1000120001000?

0010000?000?0000?0000100001011 0000?000000000000?00000?010000000001000 001000?0000000110 0???????

011?1110???1?1100?10?0000?110?00000??????????0?0???????0???????????????????????0???????????0??

00??????????????????????????????000??00???0??00???

0????????????????????????????????0?0??0000???????0?0011101?1?0???000???0000??0?01?????????0????00?

0???00?0??01?100000?00??1?10???0?00???????00???????010???????0?????0???0??0?000?00???000?????0?

0000100???0?????0??????????1?1?1??11?0?0?

Jixiangornis_orientalis

100??????????????????0010?????001????0?0??00000??????0100??????000001????0?0??1??0?????????00?0???

1?00????1??20???02??1031??11?1?0001103111000110110000111121010102120000 20101?221?10?2?11?001??1?

01[12]1101003000?00?000?0??1000??00?0100??111?001?1?????????00?000000?12300?

00010????????????????????????0?????000??00?0000?11?0?10?11?0?????0000000000?001001121001 0?0?

0000000?000???0??0????0??010?00100??0000?001000000?00??0000??00000100000?00?0000?001100????????

00??11????01?1100?10?0000?11??00000??????????0?0???????0???????????????????????0??????????0??

0????????????????????????????????????0???00010???

0????????????????????????0?????????????????????????????????0?1?00??00???????????????????????0??????0?

0???00?0??01?100?00??0?????0???????????????00?????????0?????????????????0??0?????????????????????

00100???0?????0?????????????1????11?010?

Yanornis_martini

?00??????????????????1?010?????0?11???0??2??0??????00???????????0000011???0????000?1???00??0????

1???0?????????4?1???????04??????110?011031110?011?110310????????????????????????23?010?2??10??1??

0?0121[23]0000300000??0001???00?0???????0???21?1?0??????????????????0?????300100012??????????

10???????1?????????????????0?0011?00???0210?[12]??1010000101 0?1?11?112100111??0101?0101?10000?

0?????021[01]101311[01]1100[01]100?0?1?0??0?00??????0??00?1110[01]?1?00??1?0??01100????????01??

1????????1100?11??001?11??????0???????????0?0???????0?????0?????????????????0???????????0??

0??0???0???

0?????????????????????????0???????0?0??0?0??????????00111?0?1?00??100???0000??0?01?????????0??????0?

0???00?0??01?100000??00?1??0???????????????00?????????0?????000?????0????????000?00???????????????

00100??????????0??1?????????0?1??11?0100

Apsaravis_ukhaana

????????????????????????0??0002?1?0??0?????????????????1??11??

10200??1???5?100021??24??????1?010110311000011111 0?3??000?1???1?201000?2?021?23?03?12?010?????1??

12130100?00000??20???1?20?0?????0?00??21?????????????????????????????300?10??????????????????????0?

1[01]?????1?0?????????20010?[01]001??21??1????????00000?0?1000121011111?

01011010[12]01111100001010211110?[23]120100?11??20?1[01]0010?11?110?101211211111?0000?1?0??

01101????????011??????0?1????????

0001???

???0????????

01???0?????0??0?001110001000 1?1?0?1?0000????01?010??????????0000??000?????1??????0??0??1????????

00?00100?????0??????0???????0????0000000????00000????000????0?000?10????????0?0?11????0??????????

1????1

Yixianornis

100?????2?021??1?????0?01????0?1?1?0?????????????????0100??????000?010???0?00?00120???10?01???1???

0000??1?0?40??0???0024???11?11000110311 10?001011030010002 1?101?21200302000102 3?23?0211?0??1??????

21301001 00000??000?0??1000?20??0?000001????????????0??0??0???0??0?123001000 1???????01??1????????

11[01]?????1?0?????10?20011000?11021??[12]??10100001010?111101121001111 00?0110???01?000???0?

01021[01]10?31201100[01]100?00100?00?002?101??????11110[01]?1?00??1?0?001100??????00011?11?01????

0100?10??000??1???

0??????????????????????????????????0???

0??????????????????????0??????????????00?0????????00111?0???000?100?1??000????01?????????0??????0?

0???00????01?100?00??0??1????????0???0????00?????0???0????????0?????0???0????000?00????00??????0??

00100??????????0?11????0??1??????1110100

Sapeornis

?001????????????????0?010??11000????00??21?0?00????0??????0????0000010??10?00000020??????00????

1???000????0?211??0210004??100?????211?11110100111102001011 21??00?212000?201[01]00221210????1??

01??1001210?1003000000000 0????2000??0??0100??0111?000???1???00??0???0000??

2300000000?????????????????????????0?00???000000001000010 0????????000002000 0?00000?01000100 0?0?

01010?0010000?0???010?1[01]10?11100??0[01]00?0000?0000?00?000??00?00??000001?00??0?

00001000????????001?1?1011?1?11000100000??1?000000??????????0?0???????010??00?????????01??00?

0?????????00??0??????????0?????000??0????????000??00???

00000???0?????0???0???????0?0100???????000??000?0??????0?00111?1?1?000?000???0000??0?01?0???????

0?????0?0???00?0??01?100000?000?1?10?0?0?0??0?1???000000?0???0????1??0???0?0??????0?000?000??

000?????000000100?0?0?1???00?1?????????1?1??1?1011?

Neuquenornis_volans

???????12?????????20??????????????????????????00?????

00???00??????????????????????1?11011?1031110?

011110??????????????????????????????????010???????????[01]010030??0????0??????0?00???????????

1???????????????????0???????0200???0110???????21?????

10110??0100?001?001?0??[12]??1???01?0???0?????[01]?????????2???20?1??01????????????????10?

1????????1?0???1011???????0?000???200000110???????????????????0??

0??

??00???0????????????????????????000??

0100??0111?????

0????????????????????????????????0?0???00?????1?????00??0??1???0??????????????????????????

2???????????????0?????????????????????????0010?0????????0??????????????????1???1?

Patagopteryx_deferrariisi

?0???????????????????????????????????????

0010???2??????????????????????????????????????

0?1????????????211101??????????????1???????2?010001??100121301001?000???????1????????00?0?????

1???????????????????????0?????0???????????????00?1??????00??11110000?????????01000000????????

0????????000????0101?1210[01]00???[01]?10?0???01000001[01]0001?[12]00???30???????00?2000000?

010120????0?000[12]11001100000?[01]?0??0?100?1?200??01?0???0?001????????00???0?

1???0??????

0?0??0?110????????????????????0??????????????????????????0020000??00???????????0?0?00??0001?

00000????????00111?00100???????1?0000???????0100??0?0???0?000???00????0??????00??0??1??????0?

10???????????00???010???????00000000??0?????000000???00?1???00000010??????1???0????0???0?1???10??

1???0?

Cathayornis

?0??????????????????1?010?????0?1??????????0????????????????????00?????????????00001???????

0????????[02]????????3?????2????4???0??11?02?1031110?011?1102?0?01111?0?00?12000?201?1022?

23?????????1???0??[12]1[01]1?0?3000?0???00????0??1??0??0100???111?????????0??0???????0????

2300[01]0?012????????????????????????????0??????0????0111??0010?20??1??10110?101????010?

01200010010110[01]?01020111110?[01]?1?????210120?11?01?0101011?0?000001???1?0?0?1[01]1?

0???????????0??01110????????01????????01??????????0???110?00000??????????0?0?0100?10100?

000????????????????0??????????0??00??????????????????????????????000??00?0?

0?????????????????????????????????????0??00??00?????????????????0?0100???????0?????????????????

00111?0???0?????????0000????????????????0?????0?0???00?0??01?????00??00?1?1??

0????????????????????????0????100??????0???0?????00?00???????????????00100?????????0???????????

000???11?0?1?

Concornis

??

???00?????????????????????0??11?02?1031110?0?1???0230????????????120?102??11?23??3?0???1???1???0?

1[02]11??0?3000?0???0?????????????????001??1????????????????????????????

2300??0111????10?20??1?0101100101?0?0010001?0??

[01]001?110??0102?1???10???1[01]1?[12]??????????????[01]????????????00?????0?100??1?00??1?[01]1??

0??????110?????????????????????1100110??

01??

??

0??????????00111?0?1?0?????????????????????????????0?????0?0???00?????1?100?00??0??

1????????????????????????????0?????????????0?????????0?????????????????0??0010??????????

0?????????????????11?011?

Gobipteryx

?0?????????????????1?01010?0?0??????????????????????????????11?000?0??????????1?

1???

2??

11???????????????????????????????????1????0???????????????????0????????1100100?00110?10?????

00?????0???0??0???1?????????????10??????????11??10000?0?100012000[12]001?1?0110???0?????????????1?

121??[012]0?????1?0??1????????000?0???10?001111000?1?11?

0???00000??????????0?1?01????0???0?

0???0????????????????

0??????????????????0?01000?000??00???

000??0??

1???0??0???

0??????????????????0???????????

Vorona

??

???12?

010011101001112110???00????????????????????0?????

2???

??

1010111100001?000000??0??????????????????????????????????

00???

???

???1???????00000000?

00??0?10001????????????????????????????????????110?

00000010??????????0????0?????1???1????????0

Songlingornis

?????????????????????0?0??????????????????????????????????????00?????????????000?0????

0??????????????????????????????????????11??[01]???

3???0????0?????0?????

00?????????????????????????????0???????0?????????0?01???????????????????????????????????

0???????????????21??[12]?000100?010???1?

11011??0??

0?????????????1??1?????????????????????????????

0??

???????????????????????????????????????0??00??00?????????????????0?0?

0????????????????????????????1?1?????

0??1??????????????????????????????????

00????????????????????????????????????0?1????????????????????????0???????????

Pengornis_houi

?0010??????????????10010??0?00??????0?12??0??????0?????0??????000?001????????0?0?00??00???

01??????20???0???2??????????4????1?????2?1031111?0??????23??001?1?????????????????2???2??????????

1??????211???0?000??0??000???0??0??00???0???11?1?000??????????00????00001230000?

000??????????????????????????????0?????0???0011??00100???????01[01]00?00??????0??1??012111[01]0?

011010001[01][01]1?????1??1???10[12][12]0??1??1?11??????0????00?0????0??0[01]?1?00??1?11??0?0??0?

100????????01?????00?01?1001011???1??110?00000????0??0??0?0?0100??0?????00???????????????

0??????????0??000??????2???????????????????????0?0??0???0?0?

0???0?0??00???0???????0?0100??0?0??0??1?0????????????00111?0?1000????0???0000???????????????

0?????0?0???00?0??0??????00??00?1??0?0???0????1??????????????0????1?00?????????0??????0?0?????00?

1???????00100??????????1?1????????000??????0?1?

Hesperornis

?0??????????????????00010100?00??????00021?001????00???00????1?000?001???1?0001?021???00??

10101110?2100??11?50100?210?24??11??10??00103111??????????3??0202100?1??01000???021023?03?12???

0001??11?1213110010??0?00??01?1?0000?000???00??2??1?1111????0???????10000??10?1?10?1121200110?

1010??1110011100??00?0???1010121011000012?0?000100100?0???10?0???00011??0?????????0???0????

0??????????????????????????20?1100001112110211110021111122122002?0?????00??????0001?0???0?000?

0100?11???1??01??00000??????????0?1?0100??0?????00????????0???0??0???????????0??00????????????????

00??0?????????0?0??0????0?????????????????????????????????????0?000???0????????????????????

000????????0?0??000???????0???01??????????????201??000????01??????????0????00?0???0??00?0??????

00?????1??0?0?0?0???0????????????????0?????000?????????0?????0000?????00?????????001?0???????0???

1??????????000?????????0

Baptornis

??

?2?????????5?????????24???

212????????????????????????????????????2?????????????????????????????????[12]????????????????????

11001??00??1??????????121011000012????012?0?????01?10?0???000????0?????????0???0???????????01?

[01]1[01]????????????????20?11000011121102111100211111?21020?

2???

???

?????????????????????????0???????0?0??000???????0???01???????0?????201??000????01?????????0????

0000???0????????????00??????1??????0?0???0??????????????0???????0?????????0?????0000?????

00?????????0210???????0???????????????????????????

Ichthyornis

?????00????????1220?????????????????????000111????0???????????000??1?????1000??0210??10???1?

01111?2100?1???5?000?21??34???01?100?001031110?011111023??0102?1??10212000????21023??3?12??

10011??11?1213110???00?0??20?????00????0??00????21????????????0?????????????012300[12]0?

11???????????10[01]1?1110111010010 20???101?1200111?101[12]?21102201010000 1010?

1111011211011110010110111010000101[01]10112111123120110011?12001[01]0010?1121102111100211 11[12]2?

100002?0??011?100?20???0110??1??00?00100?11??01??01???????????????????????????0?00?

00??1?0??????00??????????????????????????

0????0100?????0?00?0000?00?????????000?0???????00??000??0?01?0000??????0??00111?00100?0?10201??

000????01?0???????0????0000?0?0???0?01?1???00??0??1??????000??00100?0???001??01??2?0?00000?

00000000????0000?????00?111?0?00001????10?1?0?0?11?0??0????0??0?11?????

Iaceornis_marshii

???

????????????????????????????1101?010311??????111??3????????????????????????????????2??1??1???????

21?????????????20???1??

0??

??????21?02[23]11???1101010?11110112101????????????????????????????????1113140111011102001 0??1?1?

12110212110021??0100?11???

1??

???

??????????00111???????0?10???????????????0??0??0??????0???????????????1??????????1???????????

00????0???00???01??????????????0??????1??????????????1???0?00????????????????11?0??

0????????????????

Limenavis_patagonica

???

??011?

11??

20???

???10000[01]010101011?11[12]313011?

011??

???

???

????0?

0??

????????0000???

Lithornis

100????????????????01010100?00?111??2?02?00?0???????????????10000?00????02?101?1?????????

1????????2????????[67]?1????????????[12]??11000010311110011?11023??000210101?20200102??2102?????

12?01000???11?12130100?????0??200?01?2010???0?0?0???21???12???????????00???00000??300?11212100?

00011111010110?11[01]1101?1111?01?10121012?0111[12]1211122110100?

01011011110112101111100110001[12]1110000101010101111113130110[01]11102[01]0120110?

11211020110002111122210010[01]?0?0011?1????????011?111??00???????????01???

1??

??????????0????????????????0???0???????0?

0?????????????????????????????????1??000??????????????????????????????????

01??0???????????????

0000???11???0?

Hongshanornis_longicrest

101????????????????11010?????0?11???0????00???????????????????000?001??00????1?1?10???0??0??01??

1?0????????[234]??????????4???1??11?01110311100011111 0220?0112???01?20????????2??23?23??2??100?

1?????21[23]0100000000?000?01?1????????0?0????1?1?0????????????0????0000?1?300???

012????????????????????????????00??????1?????0?????110????????01?0??11???0?10???2100011 1???110???

2????0?????????????1??2000??000100?0??0?0??000????????1???1100??[01]?00??2?0???

1????????????????????????0100?11??001?11??????0??????????0?????????0??????0????????????????

0??????????0??0????????????????????????????????????0???0?0?

0???0?????????0????????????0???????0??????????????????01?1?0?000?0?10???????????????????????

0?????0?0???00????01?100?00??0?????0??????????1???00?????????0?????????????????0??0?0?????????

0?????????00100?????????0????????????1????11?010?

Liaoningornis_longidigitu

??

?????????????????????????????????????3??1??0???100?1????0?

21211003000?????0???

0???011?01??????????0???

0???????????????????????????????0?????01???2????????????????????????????01?1?0??0011111102 01000?

00??

??

???0?????????????????????????????0?????

0?0???00???????????00??0?????????????????????????????????

0???0010??????????0??????????????1?????????

Crypturellus_undulatus

10????012?020002?2001010100?00?111?12002100001011100010???1?10000?000??102?001?1?????????

1110111122100?01107?110??12?24??121?11010010311011011111033??001210001?20220112002 1023?23?12?

0100010011012130 1?0???01000200001?20000200?0?001021???120??01??????0????

00000103002112121[01]010001111101011001101111111110010101210101121121211112?

1010002101101011111210011111011000120110000111010101111103140101111102 0?120110?

11211020211002111122210010110??011?10102000001111110000??1100?10??01??

11??

0???

0??2??????????????????????

0??????????????????1??

0?????????????0??????????????????????????????1110?0?

Gallus_gallus

100???102?021002?2000010100?00?111?120021?0001?1000001????1?100100000000021101?1?????????

101[01]1111221?0?01007?120??12?24??1[12]1?11012010311020011111033??001211111?2?2001120021023?23?

12?0100011011??213010010001000200100?20100200?0?0???21???121??01???0??00???

0000010300110212121111122110111111111110110001101011121010112011121111211010002101111111111121001

1111011000120110000101110101111113140100121102 1?121111?1121102021100211113222001 11?0??0110001020?

000111111?000??0010110??01??

11??

0???

0??2??????????????????????

0??????????????????1??

0?????????????0??????????????????????????????1110?00

Crax_pauxi

1001011?2?021000??001010100?00?111?1200210000111000000100??1?100000000000021101?1?????????

1000111122100?111?7?120??12?24??121?11002010311121011111033??0002100?1?2??2001120021023?03?12?

0100011011?121301001000100020001?1?20100200?0?0???21???121??01??????00???

000001030021021212111112211011111111111011000110101112101011201112111121101000210111111111121001

1111011000120110000101110101111113140101121102 1?111111?112110202110021111322200110 10?0011000?020?

0001111110000?11100011??01??

11??

0???

0??2??????????????????????

0??????????????????1??

0?????????????0??????????????????????????????1110?0?

Anas_platyrhynchus

100???102?021002?2101001100?00?111?10002??00?1?100010?0???1?10200000100?021101?1?????????101?

1111221?0?011?7?11???10?2410121?1101111031111101111033??0012000?1?2??20011200210 23?13?12?

0100011011?121311?0???01000210 1?1?20100?00?0?0?1021???121??????????00???

0000011300210212111111122110211121111111011000111101112101010201112111131201001010 10?

1[01]1111121000111001100012011000010111011111112314010101110 21?111110?

11111010211002 1111[23]2210010210?0011?10?020?000111111?000??0100?11??01??

11??

0???

0??2??????????????????????

0??????????????????1??

0?????????????0??????????????????????????????1110?0?

Chauna_torquata

000??1012?0210002?101010100?00?111?100021?00010100010100??1?100000000??0021101?1?????????

1011101112100?111?7?11???1012412111?10000010311111011111133??0012101?1?2?200102?021023?23?12?

0100011011??2130100100000000200101?20100000?0?0?1021???121??01??????00???

00000123001102121[12]1111022110111121111111011000111101112101210201112111140101001010111111011121

1021110011000120110000101110101111231401011111021?111111?11211020212002111122220010010?

0011000102000101111110000?11101?11??01??

11??

0???

0??2??????????????????????

0??????????????????

1??

0??????????????????????????????1110?0?

Epidexipteryx

?01???????????????????1??????00?????0??2??0011?????????????????100?0?0????0001020???

10000???????????????0?[01]????1?1?123??10??0????0101?010?0??????00?1???????????022?????011?03?

0??????????0????011?0??0?10?000???00??1?1?0???00?0?00???0?0?????????1??2?????0???????2?0?????

0??????????0???????????????????????000?0????00??0?0??????????????0??0?0??????1???0?????????????

00?[01]0??10?0?0?0????10?00???0?????????1??11??

1????????????0?00?100?????0??????????0?????????0?????

0???0????

0???00200??02?????????0???????0?

1????????????????????????????0011??1???0?????00??????????01?????????0?????0?0?????????0????0?

0????0????????0???????????????????0???????00??????0???0????0?????????0??????????001?

00?????????????????????0???????????

Pedopenna

???

???0?

0?0??

0010???

???

????????????????????????????00?000?????0???????00????????0???11???????????????

0??

???

???00????0???00???????????00??

0???????????????????????????????0???0?

10?????????????????????????????????0?

Incisivosaurus_gauthieri

?00?00?01?001??1??10001110100 1001?010?12000011000101011010111121010000 1001?1?000210??00?

0??

??????????????????????00??00??01?0??0?0?????????100000001000102?00010?100??????0000000000100001??

0000???0000101?1100??

0000???

??01020?00???0??000?0?0????????????11?1100000????1000000?

0?0000?000?0?000???1000??1??00?0????000?110??010??01?0000?00000??00?00????0100??

0111000????????????????001000000010000???00200??02?1?000000000?????

00110???

100??????????????0??0?001????????????????????????0201100??0?

0?0??????0??????????000?0????????

Citipati_osmolskae

?001001001001??2210001011111?01011?00010210001100001000100011012112 0?010001011 1?1?????????

1011101100101211001??201??002200111?01112001101001 00?1100000100021 1001??02012020??10111111?

21010000000000?00000000000000000000110200000000?0?001000?000002011???0???

[01]0000200012200010000000001102?1??0000??000001000101 0??0000020011000[01]0?00?0010000101020??????

0????111000?0?0010000?000000000000?00100?0??0?0???0000000????00000?2000000000000000000002?000?

00000000020?000110??00[01]10?011011000000011?1100001????1000001?0?0001?11000?001???1002??0???0?

0????000?000??000??01?0000?000000000100?????100??0111000????????????????10100000000010000????

10201???2?1?0000000???0???10???00?

00????????????????????????????0?0?0???00100????000?00??0???0?00?????????0?0?0?00?0???0?

00211??????????0??00?000?0???????0??????0?0000100?0100????10?????????1?00?0????0?0?

Oviraptor_philoceratops

?00?0??????01?1????0??1??111?1011???0???1?0?11???01?0???0?11?121120?01?00?01?1?

1????????????????????????????????0????????????????12??1?????00???10??00?00??1??

0?????????????????????????????0????0????0??0?????00?00000???2?00?0????????????10000?0?0????????0??

0???0????00???0?00????????10?0?00??0??00?00?1?1????00???00?100????0????????01010?0?00?0?0????1?

100??????1100???0????????00?????????000?0??00000??????????????????????????????????????0??000?????

20????1?0???02?0??1101100???0011?1?????1????10????1???0?01????????01????????????????0?????0???00??

0?0??0??00?????00????????????????????0???1020????2?1?

0000000??1???1???

0???100????000???????????0????????????????0??0???????

211?????????00??????0??0????????????????????????00??0??????0????????????00??????0???

Microvenator_celer

??21?20?0?????????????????????011?0?

11000121100???0?1?1002????????????00?000?11000?????0010002???0000?????????????1110??0101100?

0000010???????0??0???00??11?20?0??00???0?1??0??????????????????????0???0?

2200000??0?0001????????????????????20000?000?01?????00?0??

010?00?000000000000010000????00????????0???0??0000020000000000000????????????10??0???????????????

1????????0???????????

00??

??0???00??0001?

01001?????????????010000?0?????100???????01?01??0000???00??????????????0?????0????????1????000?

10?000???0010?0001????1??????00??00?10??00000000??000?0??00000??????????1?01??11100??001?????????

0???

Caudipteryx_zoui

00010??????????????0?111??10?0001?10???21000???0?0?????????????21120?0????????0?1??0?????00????0??

00????1???0???01?????30?????0???0??0???01?0???100200?000?1??????0201202???1????101?11?????0????

01000??1?00?0?000000?1102000??00?0?001000100000?00????0???00?00000?1[01]2000000000??????????

0??????0000????1000???0000?00?0000?0?0??????????????20??0?0?000?11??00???????0?0???00?

00????????????1??0000???0000000?000000000??0?0?00????0000000??0??2000000??0????????01101100??01?

110???0??000?10???0000?????0????0?0?0001?00?????0?????????????????0???00??00??0?0??0??0?????????

000?0?????????1????0???00201???2??????????0?????????0??

0????????????????????????????1???????00??000???0000????????????????0?00??0?0???001?0?0???00?0???

0????10?????0???000??0?00?0???0?0???11??0????00????0?000?0000???0?0?????0?0?00100???

0????????????????1?10???0??0?0?

Ingenia_yanshani

?00?0?????????????????1?1?????1???????????????????????????????????21120?01000?0111?

1?????????????????????????????1???01????2?00???101112001??00000??10000010002l??????02012020011011?

11101101?0000?00011?000000000??0000??11?2000?000?0?001000???????????????????0002?0???2200?

1000000??1?????0?0??0??0000?00??1?10???000??0???1??0[01]0??02??????0101??0??0???0?0?0101000?????

1100???00?0??????????????????00????0?00?00?000?0?000??0?0?000?0?0?0??00?0????00?000000????????

0110??00?1?0?1101000??000?

1???

??

???????0011000?001?0?01??101000??0101??11??0??????00000???00?????1?000000010??1?1?0??????10???

0???0??0?000????11???0???01000000010000000??000100?000000010??????????0??1?0??00?1???1??0??0???

Rinchenia_mongoliensis

?00?0????0??????????0111?1???1?11?00010??00???0000?????00??1?12112??01000??111?1?????????????????

1???????????????0?????220??????????2??1???00?0???1??0001000?1?????1????????????????1??????????0????

0??0?0????????0??00??001??2?00???????????????0??????00????????00??210??????2??0120?????????10?0?10??

0???0?????????????

0??00???

0?00??002?0?????????????00?1?1?00001????1??0001?

0?0001?11??0??01???1????????????0????000?000??000??01?00???0?000000?0?????????100??0????

0????????????????????????????????

0??1?

1001????????????????????????????????0???????????????????0?????????????????0????????????

11?????????????????????0????????????????????????0??????????0?????????????0??????????

Conchoraptor_gracilis

?0010???????????1???00111?1???1?11?000??21000110?00????1?0????121120?010?0?0111?1??????????

01010110010??1?012?110??10????01?1?????20011000010???????0010002100010102002020??101101[01]1?

11010000??00010000010?000??000?001102?0?0000?0?0010001000002001???????0?0?0200012??????

000000000????????10???0100100????????000??0?

010??00?00??

000002000000000000?0000?002?01?????????0?020?000??0??002?00???????????0011?1?00001????1??00?1?0?

0?01?11??0??01????0?2????????0????0?0??00??0?0??01?00???0?00?000?0?0??????100??0???

00???????????????????10???0???????

0??0?

0??????????????????????????????0?0????010?????010?????0?????0???????10???????00?0???????

21?????????????????0?000000???????????????0?1?00????????00????????????0?0????0???

Chirostenotes_pergracilis

?????1??01?01101??0???1?110?0??????????????????????01010??????21120?00000201???1????????????????

1101?12????1?12???0????????????????101?1??????????00?100021?00101022120201110?111[01]??01??100???

00?100000200000??000?0?1??20?0?000?0?00100????????????????????000?????[12]????10????????001????

00????????????01?10???0????0011??00????????????????20000?

00000???00000?00??00000?00?0????????000000?0???

0000?????001020?00???0????????1????????0?001???????????????????1?0?00???

0??

??1000001000???????00200???2?1?000??0000??????????0???????0?0??00???1000?0??????????????0??0??

101000?????1?0100?0??0?00??00000?001?0??1?1?000?01?????1?0??????10?????000??0??????02?1???0110?

1???????000000000???00????00??00010?0??0?????1?0??0???????0?1???000??0

Avimimus_portentosus

?00?0???10011?00??0??1?1???????1???1?????00?11??00100110??????2?1???00?0??0111????????????

011010110101?1?00??100????????????????????????0100??????????00?211?01??0201202010?011?0?1000100?

0???00011110020???00??0?0?01?02?000000?0?001000????????0?????????00?01??0?

02001?????????????????????0??0???00??0??????????02001000??????????????????????????????0?????0?0?

0010000?000000000????????????2???????????[02]0?000?0000020?010000000000000??00?01??0??????

0??????????????????????????????????1?

0???

100??0?1100????????????????????0000001?0?00????????????????????????????????????0???????

000100000l??????????????00001???????101001??0101?0100?0?000??001000??00???????0??0?1001??101????

0000100000?????1001101102?????10101000000????00?000???0010000000000010?0????1??01????0??00?1???

1??????0??

Falcarius

??01?1110000111101 0?????1????00???????????00??????0000000??????100?000??0???????001010001?1??

000111010121010001200010 0??0000??????000001000100010?0000100?

0110010102203010101001000100000000000000110000000000000??000?10?00?0?000?0?001000????????0?001???

00?0?0?0??11?0??000???????000??00???0??00?00100?0?00??0000??0010000[01]0??????????0100020000?

00000001110?1?0?0010000?0010000000000010000???0100???0000000?000000000200000000000?00000000?

0000??00000?0?200000??0???00000?1100?00000000???????????????????000?00?00100????

0?????????????????????????????????0?????0???????????????01?00???00?00???????????????????????

0000001010010????0??00000001???0???????????????0?0???????0001000001000??1000??

1010000000100000000000?0101?0100000000001?0000001000?0??00101000010?1?01?11?

100010010000000000111010 02?0?101111000000101000100000??000001000?000000?0?10?10000?011011002?00?

10?0000???

Beipiaosaurus

0??1???10?10?????????????1001001?100????

1??????????????000??0220?????????0000??0????0?1000000?0000??001???20220??0??0????0??00?0??

00??????0000????0???0??00111???0?0??0????0010?0??????????0??????????0???0??????

0?????????????????????????????????????00???001000??????????????00?

00????????????????????????????????10000???????????1??0100???000000???0??0??0?????0???????

0000000000???0?0?00000????????????10?????1?1100??0??000?1?0?????0????????????????????

0??

?????????????????????0??0?00?????????????????????0???????0??????????????????????1???0?00??00???

00?1????????10?????0?0?????????00???????1?1?0????????1??1?????10???000?0?00???????????????????0??

0?01000?01010???0?11??10??0?1?0???1???????????????????1???00?0?0?

Segnosaurus_galbinensis

??21??10?0000?000???0100?001????????

1??????????1?0?0??????????????????0?10?1?00000????0???20011100102020221??0110201101?11001?00?00?

11000000021?0??00?01?1??0?0???00?0?010?00??????????0?????????00???????

0?????????????????????????????????0?0????0??????????????????????????????0??????0????1????1???

0010000?00100000?????????????????????????00?00000??10??????00????000000000??21?0????

00???????????????????2??????????

0??

???

???10????????????0?00???????21020010?0101??02???????????0?00??????????????0??01??0??1??1????????

2???????0??110????????1???????10100??1?11122210111111112?111201??????????0???1??????????????????

1?

Erlikosaurus_andrewsi

?0012???2?0?1?1??1010011100??0001?1000001000010000000000??11112100100000020001?

001001001???

0???0000?021?0??00??11??00?0?00????????????

0?00000010010??00000?000????0?000002000001???10?0100???0000?0?10000??

0000??1?0?0010000?

001000000??00000000????????0000?0????000?????000?

0?????????0????01?0001100????1?10000?0?00?0?10000000???0??0?00??00?0????0000001000000??00?0200?

00000101000?00001?000??00?0?00????????????????00000001101????0???0100100020000000000000??????

0?????????????????????????????????0?00??0000???????00??

0???0?011????????2??????????????11210?11???????1?0?1????????????????????????1?01001000????

01??????????010?0??????1?

Alxasaurus_elesitaiensis

??210?100????????????

1001001??????????0?01010000?1?0101002?0?1???????????0?000??0?10?0000120?11??0??1?20?21???

11?????????1????0?????????00000?10000??001?1??00?0??00?0?0???00??????????0??????????0?????0????

0???????????????????????????????????0??00????0??00??[01]0????????????????0????????0??11??01??????

0?0???010000???????????10?0100???000000???0??0?????????????????????????????100000?0?????????

0?????????????????????

000??

??0??0020?????????????????????0????????????

11101?????1??0?11??0?00??010?0?????01?????????2????????????????200??0????1?1??0??0????1??1?

100???????0000000??1??0???0?00???11?0????01100??11?1?????1111?????1?00???10?1???0????????????

1???????0???

Neimongosaurus

??21????0?????????????0??001?1??000?

110101?1?0?10?2?1?1?02200????????0001010000??????????????111001?2?????????????????????0??00????

0??000000021?00????0??1??00?0??00?0?????0???????????0????????????????1[12]200?

0?????????????????????????????????????0?????00?0000?0??????????00000200?0?00000?0?11001?0?0?10?

0???010000?0?00?????????????????????0???????????20?00???????0?00000000?0210??????0????????

0??????????2?1100?

000??

??0????2??????????????????????0???????0??

1??11???????0??01?10?0?00???????21?2?????????????2?1???1?00??00000?1???0??00????00????00??????

100010?2???????00?????1???0?0111?111001011????1?1121?????111111????10????1??11??0?0??0?100???1?

1?????????

Erliansaurus

???

??10??1??????????1???????????????0?0???10?0?0???00000????1?0?1??????????????2??1????10?00?0?0001?

0??00??????????00?????????????????????0?????????????????????0??????

200???0????0????????????????????????????????????01?0?0?

10?0???010000???00010???????100???000000???????????20?00???????0????????????????

0??????????????????????????????????

000??

??

0???????????????????????????????0?0?1?0?01????????????????02?0???1?000?????????????00100???????

0?????????00???100?????00?11????????????????011?111111??11?1???0?1?11111011?0?????????1??????????

0?1??????00?0???

Suzhousaurus

???

?0101??01000??0??1????0??????????001010000???????????200111001?2020?2?10010020110101100?

00??????????????????0???????1????????00???010???????????????????????0??????0?

0???20010000[01]????????????????20000?00000?01010?1?0?

0010?0???0100000??00??????????????????????00?000?00?1020000????????????????????

0??????????????????????????

2??

???

?????????10111100100000?11010?00??????021020010?0101?00201010????????????????????0???0??01??00?

1????1000?0?2???????00????????????????11?100011????1112212111110??????????????????1?0??01102?0?

2???????????1??

Nothronychus

?????1112??11?1?0?0????????????????????????????????00000??????????????????????????????1??????????

10101?10?01?1?00?100220?001?????00000?100?00????00000200??10?1?2020221100100201101?1100100?

100011000000021?00???00??????0?0?000?0?010?00?????????????????????0????1[12]200???????????

1?????????????????????????????????0010000[01]0??????????00000200?0?00000?0211001?0?0?10?0???

010000???00010???????000???00???00?000000?1020??000000000?00000000?0210??0?000?0????000????????

0???1100?000?

000??

??????????????????????????????????000?01100???????????????????????????????????????0???????0?0??

1010110???0000?110?00001?0?0?021?20010?1101??02???1?10000?0?000?10?????0?1?0000010?0??1?11?1?

0010020000?00001110?1?2??????1?1110?0001???111122210111111?12101120??0????1?1?0???10?????1???

10????0???

Enigmosaurus

???

??111?01?2020221100??

0201101??????????????????????????????????1????????00???

010???

???00?000000?

10???

2???

??

??????????????1??1??????????????????????2?020?10?1101?????????????????????????????????0??01?????

1??1?

122121100??

Nanshiungosaurus_brevispinus

??1???????

101?121???

0???

??0?????

1???200??

0???

0??

2???

??

0??????????1???1??0????????????????????????21?2???10???01????????????????????????????????????01??

0??1????????????????????????????????????1??????????????11222??

11???

Therizinosaurus

??

?????????????????????????????????0010?0000????0?00200??????????????????????????????????????0??0?

11000000021?0????00??

00??0??????????????????2000??00000?01?1001?0?0?

10?0???010000?????010???????100???00000??????????????????????????????????????0??

0100????????????????????????????????

00???

???

??????????00?1010?00100101??001?0????????0??????????10??

0000????????????????????????1110111111????????????????2?111?0?????????1???01????0????????????0???

Haplocheirus

?0010???00010?0?12011011101112001?000020100?0?1010??0001?0010000010110110?1000001101010100??10??

0?0111201??0?00001?0???0?00???????10021000121 0?0?00010???001?00?10100201001 0?011000?00000000?

0000?0000000??00?1110001100010001?0???0010?0100000000??0000001?00000?102000000000????00???00???

0???000000??0??0??0000?000?000????0?????????????20000?000000010101??????100???000?00??????????

0???00000??0000000?00?000100200000???????000?0??0???0100?000???0?0???????????00?0??????????0?

0000001?00001??0?0000000?0?0100000?0??11???0??0?01??01?0????00000100000??00??100?00000000000?

000????00??0??0?0??????001????????0?0??0?0000????0???00000??00?00000??00??????????000???????0??

1???????????00?1010100??0100?00?0????????0?01??0???00?0?????0?????00000?00?101000??00?0??010?

110010010001000001 10??00??0?101???00000???0000?????????000????????00101???0?1?1?010?????0??0000??

10000???

Alvarezsaurus_calvoi

???

01000???0???0?20?0?2012?????????????000?00????????0??1?0000101?00?2???????????????????01?000??0??

0??11000000??00????????1???0?0??00?0?????0????????????????????????0????

10??00100???0????????????????2?000?????0?0?

0??0??000?00???2????00000000?000???00?02???

0?????????????0????????0??????????

00???

??0???????0001?

00????????00?10?0?0????????????000?000?????????????????0??00?????00????????????0??0??0????????

1000???????10?????101???????1????00?????????00000???????1???0?000?10??????1???0?0??0??

0????????????????

Patagonykus_puertai

???1?

01?112????012010?2?2??????????????00210???210?????1?0???1????1010?????20????21202??

1100000000000001210??0????001??0???????0?????????0????0?????????????????????0?????

01???0?00100???????????????????20000?000?00??????0?0?

0000??0?0010000???0??????????1????????????0???0????10020000000000??00?0???????10??

0???????????????????????????????????

0??

??

00001?01100?0?????000?1100??????11???0?0?01?01?1?000?10??1????0?00?????0???00?000?0??1???0??

1001?101??1?0?1111111????????????0?00000??0?????00??0000?00?0?00??1?????????10??1???0???0????1??

0???0??

Achillesaurus

???

???????????020???2??????????????????????????????????????10??01?2???????????????????1??????0??1?

01000??0???00????????1???????????0?????0??????????????????????

0??

??0???????????2????00000000?0??0??

0???

??

???00?0??????????0????????

00?0????0???????????????????????????????11????????????????11??????????????0????????????000???????

1???0?00?????????????0?????0??????????????????

Mononykus_olecranus

??????00???????112?????????????????????????????????????100????????????????????????2????00???????1?

1?11102001???2?10?2??????1???1000?000200030210?002?1100??1????1?201000?2?????3??0??

02101001110112120000300000001??00???1??0?0??00?0?0???00??????????????????????????

00011???????????????????????????????????????0?0010?0?????210???00?????2?010?0?0?0?010?011?0?

0001000?001000000000??0000???30000???0???0??00???????200000000000??0000??0?00?0??0??000???????

01?0?????0?0????????

11102???

??0???????

1001001000????????1010100000100?????011???0???01?01000001010 1?100000100????0??????00??1??0???021?

111001011111111101011???????0100000000000?000?0000??000010000?000?10????????100?10100?0?01???1??

0??0?00

Shuvuuia_deserti

?00101002?01011112011000101 1?01111?1002010000101001 0010???1110?0010100100210 0??0211??000??

0111111011102100?0?20101201211010 0?1000?00020003021??002?110000101001?201000?2002203??03?

021010011101121200003000000110000011000000010000?00??001000000001?010??000000000?000100000000?

101000?1000011 0?0111000??0[12]0????0100?0010000[01]0?0210?0?00?????20010?000?010101011?0?0001000?

00100000?000?????????30000??000??00?000?00?10200000000000000?0000000?00100000?0000020?0001?01?

1010001???????1110200?000?000??0?1??0000?0?00000?00000100???0??0?00??01?0????0?????0??000??00?

0200?000000??0?0?0??00?000?001?0?0???????????????????000000000????????00000?000?0??000000??

00??????0?0???????00010010001011?0001010100000100??0?01110000???01??1??????????0???0????00001????

1??00??1??00110210?110010?1111111??0?01002?0??00??0?0000000?000000000??000?????00000010101?0??0?

00110????00?1101?0????????

Parvicursor

???

???10?????????1??2??1????2?1000???02203??03??2??100011?

11211000030??00?????????1??????????0?00??0??????????????????????0?????

0??0?00?

00???0????????1?

2000000000000??000?000?00?0???????????????0???????????????????

11???

???

?1000?????00?????????????????????????0???01??10??00?0?01??000??100???????????00??1????????1?

111???????????11100010????????????0????????????0?????000??00000000010??????????0???1?0????1???

1?????????

Albertonykus

???

??211?????1?

0??030?????????

0??

???

??0??

02???

???

????????????????????1??????????????????????????????0???00????0??????????????????????????????????

11???11??1???0????0???????????1??????????????????????????

Albinykus

???

???0????????????????????????????????1101??

121100300000?????????????????0000?????

0??

???0??????0????????000000000?

0000000?00??0????00????????0???????????????????

11???

???

??0?010000??00???????????00??

1???00000?10??????????

0??????????1?????????????

Bonapartenykus

???

???112??0????????????????????????000210???????????????????????01????????????212?2??

1????????????????????????0???????????0?0???????0????????????????????????????????

0??00100???????????????????20000?000?000??

10???100??????????????????????????

0??

???

?????????????????????????????????0?1000??1?????0?010?010??????????????????0?0?

01?????????????????????00?????00????????????0??????1???011??????????????????????????????

0??????????????????0?????0????????????????????10????0????????????????

Ceratonykus

?0010???2??10??????????????????11???10?201?0?0?0100??0??????????????01?0???????????????????????????

1?1????????????????????????????10????0?2???0??1????????

212?0003???00?????????????????????0?????0????????????????????0????????????????????????00???????1????

111??????????????????????????????21?????0??????0000?0???01????????????????????

0??00000000??000?000?00?0?????????????????1????10?

0????????????????0??0???????????????????????????????????0??????????????1?0????0?????0??000??0??02???

0?0000??0?0?0??00?000??0????0????????????????????0??0?00??????????0?0????????????????????????????

0???????0001??0?0??1000???????

0???????????????????1?????1?00?0??????????????1???00?

00001????????????01???????????????????????

Linhenykus

???111?

101?1??1?0???2010020?2????????10?0???1??????????0?24110??????????2??????????????????0?1010001????

2?2?0?030?0000???0????1???????????0?????0????????????????????????????

01???0?0012?0?????210????0?????20??0???0?0?

0????????????????????10000???0???????????31??????00??0?????????1?2000000000000??000?000?001?????0?

0?????????1???????0?????????????

02???

??0???????0001?

01000?????0??????1????????????????????????????0?0??00?0?0???000???00?0??????1??0?????????????

1011????????1111?1???0???????????????0????????00???????????01??00?00001???????????0?1?110?

0?????????0??0???

Xixianykus

???

???102?00?21201??0?????????????????????????????????????0?01?1??1?200000?20022?3??03?02?

010011101121211??3???000???????11???????00?0?00??0??????????????????????

0???0?

0010000???00?000000?

1?2000000000000??000?000?0?????????????????????????????

0??

???

??????????1000?0??????????????????????000110000???01?010??00?0?00010?0???????????????????????0??

1???1011?????????????111??11??????????????0???????????0?0000??00001000000001??????????0????100????

1???1?????????

Nqwebasaurus

?0?????????????????02????2010????????0100?00??1?????0?00?????????????????????????02????1??????00?

11001???1????????????????????????????0102?000??0?00?10110???????????????????????????00??????0?

00001000100000000?000?1??00??????0?0?0?????0????01?00???00?????????????????

10200??????????????????????????????00??????????????????????????0??????????????20????0?0???0101?

0??????10?0????????????????????????00000??00000??????????00?????????????00?0????00??0?0??

00000????????0??????0?0???????????0?001??????????????0?0??????0???0??????????0???0???????

0???????????????????????????1???????????????????????000??0??0?0???????????????????????

00??0??????????????????????????000??01?0?1?0?

0?????????????????????????0????00?0???00?????0?111?0???1??0??012?????100??00010000100001???????

0?????????????0?????????????????0?000010??????????0???????????1?1???00?0???

Shenzhousaurus_orientalis

???0????????????????21000??10?0??1???000??00?000?0???????????0?00000000??0??0?1?12?0??

1????????????000??1???0???00000???0???????????????????????????010000000??1100010010 1?000001100??

00001?0????????????????????0??1??1?0011?????00???0010??10000010????????00??0?00???0???00?

000?????????????????????00?????0??????0?00?0?100?0?0?

0??0?????0?00000?00000?0002?

000???????????????????????????00??????????????????00000???????????01?00??00000??0?0000?00?0?

0000000000??00???0??0??1???1??

10???????0????????????????????00?00??00?00?0????0??????????????????????????000???????

00???????????????????0101000000?0001?00??0??????????????????01?????0?10??00??1??00?2?0000????????100?

0????????00?10????00????????00000000???00??????????????0???0??0???1????????????1???0???0???

Ornithomimus_edmonticus

?00010?1101101?10102100 0?01010101100000000000000001000000??????0000000010?1001?1????????0?001?

10110000111000101000000000100100??????01120012000000?

202001000000110001101011000001100100000110001000100000020??00?0111111101211100 00?0?

001000100000000 1?????0001?000000002100000000?????????0?0?10???000010110??????000000010000[01]0?

0??????????????0??0?0?0?0?01?1001?0?00?000???0000000??00??????1??00000??000002 0?000?

000002000000000000?0??0??????0??0??000?1????????0???0??0000000????????0001000 0?00000?00?0000?01?0?

0100000??0??00???0??0001??01?0????00000100000 0??01?00????000010 1?0000??000000??0????0????????

1??????????1????????????????00210???0?00000?010?????????????010?00?000010 0011?????????????????????

10?00???1???????????????????????????1????????0111100111111111?11000????????100??0???00?01????101?0???

1??0?0000?10000000000????00000000000000100?0?????????1????????????10?01???0???

Archaeornithomimus_asiati

??00?

1011000011100001?100000000?0??????????010200?20000?0010[12]0???000?

01100011010110000010201000001100??000?0000020??0000?10??10??1?1??00??0?

001000??0?001000?

[01]0???????????????20?00?00000?0?0??01?0?0010000?000000000000010000???00000??000??00???

00000002000000000000?0000??00?0?10?000?????????????0??????0?0????????

00001??

??0???????0??1?

00001?00??00000?00020010?010?010100000?01011?00000000000?000001000????001?101?001?11?0????

000010000001100001??011???????110?000001100000000000??000000000?000?10??????101?0?1?000?000????

1?????????

Anserimimus_planinychus

???

?????1?????????????????0??????????1?200?2????00?20200100000011000110101????001?

001????????????????00002???00????11??10??1?1??00?0?

001000???

??????????0????????0??1?????????????????????????????????????01000??00000?????????????????????????

10000??00??0????00?1???????????????????????????????

001??

???

??????????????????????10?0??1?1?????????????????????????1?0??????????11011011001??1?0????????

100??01?00??????????????????????000?10?000?????????????????????010????????????????????????????

0000???

Struthiomimus_altus

?00010?110??0??101021000201110 1?110000000000000000010??0001?01??000100000102000 1?1?????????001?

1011000011100010100000000100100??????0112001200000002010010000000110001101011000001?

0010000011000100010000020??00001111111012111 0000?0?001000100000000 1??????00?1?0000??2210????

0???????????0?0?1????000?????0?00???00?0000?0000[01]0?0??????????????200?0?0?000?0101001?0?

0010000??00000000000?100000??00000??0000000?000000000200?00000000000000??00?0210?0000?

10022000000?0??0?00?00?????????0010000000000?00?0000?01?0?0100000000000???0??0?01??01?0????

0000010?0000??01?0000??0000101?00?0??000000??00?0?0???????????????????1000011010?00????00210???0?

000000000????????????00??00?00001000011?????000000000200?110101?10100100?01011?

0000000000000100010000011100111111?1?110000??00001000000110000100011010 0???1??0?

000011000000000000???000?000000000100?0?0?101?1110000?0000?1010???00???

Gallimimus_bullatus

?00010?110110101010210002011?010110000000000000000010000001?0100000000000102000 1?1?????????

001110110000111000101000000001001 00??????01120012000000??

2010010000000110001101011000000110010000001100010001000002 0??00000111111012111 0000?0?

001000100000000 1?????00011000000002 21000?00000001000000?0000??00000?0?10200??100000001 0000[01]0?

0?????????????20010?000000001010 01?0?0010000?000000000000000100001??00000??0000020?

000000000200000000000000000000?000?0210?0000?1002??0000?00??0000000??????0000100000000000??0?0000?

01?0?01000000000000???0??0?01??01?0????00000 1000000??01?0000?00000101000?000000000?000?0?

000????????????????0010000111100000???00210?0?000000000000000????????000000?

00001000011[12]00000000000000020011?0101010100100?01011000000000000000100?1000111011111011001?

110001??00001000000110000100011010 0???1100000001100000000000??000000000000000010000?

001010111000010000?1010?00000?0

Garudimimus_brevipes

?000?????01101????02?00020101000??000000000000000000?00001?01000000000000002?001?1???????0??

0111????0001?10001?100??00?????10???????????????????????????0000001100????????????0??001000001?

000?000?000001000000?01??1110?2?1?0?00?0?0??00010000000?????00001000000010???00?0000?001000000?

0000??0000010110000??000000000010000???

0???00?

000000000200000000000000000000?021?0????0000???00???0??001000????????00???0000000000002?0000?

00?0?0100000000000???0??0?01??01?0????00000100000??01?0000??000000000000000000000??00?0?000??00?

010????????0010000000010???0???00210???0000000000000000????????0?0?00?0000??

000111000000????????????????010100100?0101?0000000?000000100?10001111?????001000?1?0001??

0000????????????00001011010??????000??????????000000??0000000000000010000?0010??01??0011??00?111?

0?0???00

Pelecanimimus_polydon

?00???????1?????????2100?2?1??0000??000000?0000???????????????0?000?0000??????000211??0001??000???

0?0?111?00????????????????0??0?00???120????00??0?

2010??1001010????0?????0?????0????10000??0?

1?0????00????0000?0?10???0?0???????????0?0??????????????????????00???0?10??????0????????????????0??0?

0?0???0?????????????????????????????????1??00000??00000???

0??000????????????1?????0?00????????????001?00??00010??0?0000?00?000000000000??00???0??0?01??01?00?

0?0000011??000???????????????????????????00??0?000???????????0010000????0????????????????????0??00??

00??????????????????00000000?10???????0?0????????????????????????0?

01??????????????????????????????????????010?1??111??????0????0?2?0?????000001100????????????0?

1101???????????0?000????????????????????????1???0??????1??????????111??10000???

Harpymimus_okladnikovi

?0??????????????????2100??????0??????000000000000??????????????000000?0000????1?1200??1???0???1???

0?00??10001?100000?00?001????????0?1?0012000000000010?10??00???????1?????????????00?00??????0??0?

010000010??000001011????1?0???00?0?0???001??0000??1?0????00??0000??11?1000000????????????

0??????????????0000??00?00000?0000[01]0?????????????????????????????10??01?0?0010000?

000000000000???0?010?00000??0000000?0?0000?00?????00000000?00000?00??11??0000?0????????0???????

000?????????0?001?000000000????0????0??0?0?????0000?000???0??0?01??0??????????????????0???1?

00??????0??????????????00000??00?0?????????????????????????????????????00210??000????????

0????????????0???????000??0000?1??0????0000??2001?00?01?1??0010?????????0??????????00?0??

0001100100011??0??0?11?00????000???000001000????011???0??01???00000110?000000000???0?00???0?000?

100?01??1?1?01??00??00???1????0000???

Beishanlong

??

??????????????????????????????????010200120000????0100?????????????10???1??00??????000001100?

10001000?01??000?0???1???0??1?1????????01000????????????????????????????

210???0??0????????????????20010?000000010100 1?0?

0010?00?00000000000000010???????????????????0??????????2000000000000?00000?00????0??0?0?0?????????0?

0????????????????00?

01??

??

?????????0000002001 1?????????????????????00?????0?00000?0???00?????00??1?0???0??1??????????

1000000?1??00?0?011??????????????000100??00?????????????0000000001?????????1???10?0??0??0???1?????

0???

Sinornithomimus

?0001?????1?0????1?21000??1010001?000000000000000000?000??0???00000000?00????1?1?????????0001?0?

10?0???100?1?1??0?00?0?0?100??????010200120000?0010100100000011000110101 1000001?00?

00000110001000?000?010??000001011100121?1??00?0?0010?0100000001?????00001000000?

0210000000??????????????00??000001?1?0??????0000001?000?0?0??????????????2001 0?00?00?0101001?0?

0010000?0000000000??0100001 0?00000??0000000?000000000200000000000000000??00??110??0000?????????

000???000000????????0?00100000000100?00?0000?01?000100000??0?000???0??0?01??01?0????000??10??000??

01?0000??000??????00000000000??00?0?0?0??????????????????10000?????????????00210???0?

000000000????????????0???00?00001?000?????0?0??000000200?100?01010100100?0?010000?000?00?0??1?0?

1?00111?1001110?1001?11?00???0000100000001100001 00?11??00???1100000001 10?000000000???

000000000000001000??0?101?011?000?0?00?101??00?0???

Qiupalong

??

???00??1100????????????01100?????????0??

000100000 2???00????????1????????00?0?0???

0???

0??20?0?0?

00000?????00000000?0000?000?02????????1????????0??????????0????????

00??

??

??????????????????????????????10100?00?0001??????????000?010001??0??????????1??110????

0?????????????????????1??01???????????????????????????0000??000??0000?00001??????????01??????

1???????1?????????

Huaxiagnathus_orientalis

?00?0???????????????0001???1???0?????00??0??????????????????????00?0010????????00010?1010?00??0???

1????????0??????00???0?002010????000000000?00??00000000000 20?0??000100201?000012?0???0??????0??0?

010000000?0?011??000????00?0??00?0?0010?0100000?0?1?00????0????0000??200???

0000??????????????????????????????????00?00?1??0?[01]0?0?????????0000020?00?0?0?0?010100??????10?

0???0????????????????????010????000??00?00??00000??????000?0??00??0??0????10000000????????000????

00000?0?00??000000?01??00000??0?00?0?00?10000000000???1??????????????????????????00?

00????????????????????????????????????0???02?00???

0?????????????????1?0?00???????0?0??0000??????0??0001100???1?0?000??00000??0?????????????0?????0?

0???0?0?0?00?101000??01?0?00?0?00001000?000??0???????0?????10?????00????0?0?0?0000????0???0????

0000100??????????????????????000???00?0?0?

Sinosauropteryx_prima

000?0??????????????0001???1???0?????0000??????00??0????????????00??01???????1?0001001010?000?01??

100????1?0?0????00?110000201??????00000000?10??000000?00020??????0100201?000?11?0?1?00?0??000?0?

01000000000?011??0000??000?0??00?0?000??01000000?1?00?00????000?0??20000?000??????????????

0???????0101????????0000?0??0?00[01]0?0??????????????0?00?0?00??010?000????10?0???00?

00????????????1??0100???0000000?00000??0?2?????00??0??00??00?0???010000000????????000?10???

000?????????0000?01???0000??1??1????0?1?0?00??0??????0??????????????????????????00??

0???????????????????????????????????0??02?00???

0?????????????????000100???????0?0??0010??????0??00111?0???110?000??00000??0?0???0??0???0??0?00?

0???0?000?00?11100???0000?00?0?0?001000?0?0?0??????010????1100???0000???0?000?00000??000???????

0000100?????100??????????????000???0000?0?

Compsognathus_longipes

?00?0???????????????00101?1??00???00000???000???000???????0?00000100?1?001000010010101000?01??

1010???1?0?0???000?0120002010?????0?000000?10????0?000000??????0?01002010000012?0???????????00?

000100000000000001100?00????0?0???00?0?0010001100000001?00000000?0?000??020000?0000??0?0??0??????

0???0000????01?0??0000000010000[01]0?0????????00?0?20000?0?0?0?010100????0?1000???0???

00??????????????010????0?0??00??0?000?00??????00??0?0000?00000?0010000000????????00????

000000????????00000?001?001000?1?0100?0001000000000000??0???0??0?000?00?0????0000000?00?0??01?

12????????000???????11?000??????????????????????????0??????????????????02000?000000200000?0???????

1?0000???????000??0000?0????0????????01???1????00????0??0?0?0???0??????0??0000?0???0?000000?

101000??00?0??0?000?0010???0?0????0????10????1100??0000????0???0?00??????00???????0000100?

0010100??1?0????????001???00?0?0?

Juravenator_starki

0000????????????????00010??11000?1???00000?0000?0?????00?????0?0000010???0????000100101??00??0???

1???????0????????00??10000201 0????000000000000????0000000000?01100???????????????????0?????

00??????0?0??000000?10?000?0??0??0??00?0??????01000000?1?00?0?000??0000???20000?

000???????????????0?????????????0??????000?00???00??0?0?????????[01]00002???0???????010100?0?

0010000??000?000?000???0?0????100???0000?0???00?000??2??00??????000??0?0000?0010000000???????

00???00?0?00???00??0?0000?011000?00?01?0100?00?1?00000000000?00???0??0?00??00?0????0000000??000??

01?10????000??00???????11?000??00?0?????????00?????????0??????????????????02000???0?????0???

0???????0?010???????????????????????????0000??00??1?0?000??????0????????0??????0?????0?0???00000?

0??101?00??00?0??0?0???0????0?0?0??0?0?????0????0?0?????000???0?000?0???????0??????????00100?

0?????00?1??????0???00????00?0?0?

Sinocalliopteryx

0000????????????????0001??0???000???00?000?000??0???????????0?0?000001???????1?000100101010000?01??

10?????1?0???????00??100002 0?0?????0000000??00??0000000003?0?0??000110201?000012?0??????????00???

0??000??0000?011??000?1??0000??00????010?01?000000???00?0000???00000??20000?0000?00?????0????

00?????0??????????????00000001000?[01]0?0?????????00?0?20000?0?000?010100??????10?0????0??

00????????????1??010????0000000?000000000 2??00?0????00?0??00?00??010000000 0????????00??10?00000???

00??00?000?01???0?010?0?0000?000??0000000000???0???0??0?00?????0????0????00?00?0??0???2?????

00????????????11?0????0??????????????0?????????0???????????????????2?00??00?00????????0?????1???

0000??0??000?00010??????0??000010????1?0?000??00000??0?0???0??????0??0??0?0???0?000?00?100000??

01?0?00?0?0?0010?0?0?0????0?0???0??0?0?00??0??0???00?000?0000???00????????000101?????10???

1??????????000???00?0?0?

Mirischia

???

????????0??0?0???????????????????????????????????????0????????0001?02000???012100100010110????

0?????????????1?????????????????????

0010??0?1?

0??0???00?0?

000??

???

??0???000??????????????????????

1??????0?0?00?000?1??0?0??????????????????????0??????0??0????0????0???????????

1????????????????????????????000?0????00??00??????????????????0??????????0????1??????0??

Ornitholestes_hermanni

?0002???0?0?00?1???0?010?01110001?100101?0??1000001?01011????0000001000?0?000010?00101001?????
011?0101210000??000?0010??1???????????????01?00?????????00001??1?0?0100101000001??0?1???????

0????????00?00????00000?0010?00?0?0000?0?0000?0100000000001?01?00001000002?112000000010?0??000000?

0?00???0000?0?0000???000000000010000[01]0???????????????????????????????????0?0?0010000?

000000000000??0??0????1000??0000000?000?00000?0000?????????0000?000?001??00?0?????????00??0???

0001?0??????????00??00?1?00000000000000000?000000000??00??0???0000?000?00?0????0000000?0000??01?

0000?00?0000100??00001?000?0001100?00000?01000?????000000000000???0???020000000000020000?00000?

00000000???????0001000002?10?00?0??????10010?01??000000000???0100????000????0?00?0000?

0000?????????????001??1010?0000???0???0????0?00??001000000?00???000000???000000??000??????????0?

1000?1001?0001????0??0?1010?01???????

Coelurus_fragilis

??0??

01001000012100 0?????0?0002?????????????0?0??0?10001????0???????????????????????????01100?000001?0?

0000110?0?0????0?00??00???0??????????0?0????0??????????????????????????

11200???001000??????????????????2??????????????01000?0?

0010000?00000000000000010000???0????????0000?????????00200000000000??000???????????0000???????????

0??????0???????????

0???

??0???????

000010000 2?????0?000?0??100100?????????????0?0001?000000000?0000?????0?????????????1??00??????????

0000??000???0??00?0100????????11000000000?00?????????00000??00000001?????????0?1?0?

11221111?????????????

Tanycolagreus

?000??????????????????00?0??????0???00110????????0?00????????????????????????10?00??????

1?????????????0011?000?????0????0?0??0???????00000000011?1000000????????????????????????

11001000001100000001000000000000??00???0??000?????0?0???00???????????????????1???1????[01]2000???

0????????????????????00??0?0???????????0?00001000??0?0??????????????20000?000000101000?0?0010000?

0000000000000100000??01000??00000??????????0020000000000000000000?00100?00000????????0?

0??????????????????00000?????001000????????????????????0??????021000000?000?0??????????????????0??

0??00????????0?0?000??00??

0???????????????????????00001????????00110010010000000??????????00???0000000000000000000?000?????

00100000000??0??0?0?100?1000000000000101000??????????????000000000?????????0?000000000010?????

0??0?1001012210111???1??0??1???

Tugulusaurus

???

???????????????0??????????????????????????????????0?0?????????????????????????????000?0?100??

00110?0?1????0?????

0??

???

?????2000000000000??

01???

???

??????????????????????00?????????????????000000000000?0???????0?????????

0???????????????????????????000001???1????????????????????????????????????0?????

00??????????????????0001??1????1??0??????

Zuolong

?0000??????????????0000010111?0???000?0000001100000?????1000?????????????????00010?101??1?

010001000????1???000000?0000??????????????????000?0??????0?????00?0100?????????????

01100110000110?00?0???00010?0?00?????0?0100??????00???0???0011000000??0???00??1???0??0002000??

0000?????????????00??0000000??????????010?0?10??0?0????????????????????????????????0??????10000?

000000000000010000???????????????00?0?0?00?0020000?????????0000?000?0?1???0??????????????0???

000100????????0??00?????0000000000000?00000001000???????1???0??00?0000??????????????????0?????

0000?00000?0?000??10??0010?0001101?00000??

0??????????100???????00000??0???000???0??????00000?????0?00000?0?0?0??0000000000????000?000?00??

0????0?????000??00?0???0???????????000011?????0?0??0000?0?00000??00???0?0???0000000????00??0?????

1?0?10??000??01?0?01?????????

Bicentenaria

???????????00?1??????0?0??????00??00?????????????00?????????????????????????0?

000000001????????????100?????????0?????????????????????0?00???????????0?1????0??

10?????????????????0???00000?10???0?110???????0??????????1????0??????????????????00??0???

0???????????????1???0???????????00????????0??????00???????????0???0010????0????????????????2???0???

0???0?????0?0?001??????0000000?0??????????????????????0??0???0????2000?0000000?????????????

0??????????????????????0?????????????????00????????0????????????0???00??

0???????????????????????????????0?0000000???????????????00000000?

1???1?2?00??000??0??????

0????????????????????????1?00??0????00????????0??0?????????0000?02???00?0???????00????????0?????

00??????0???00???0????????00???00?????0??????00??00?0??????0??0?????00?????00???????????0?????0?

0??0?1?0????????????

Kileskus

????????????????????0?01120111???00000?

101???

???????????????00?00??0??????????????????????????????1?0000?0???0??00??????1????????0??

001???

??

????????????????????1011201???000?0000?0000?

0???

?????????????????????????????100010?????????????

00???00?????00???????????????

1??????0?????????????????????????????0???

1??????????????????????0?????????????

Guanlong

?00020?000000?010[01]1000112011120 00?0011000000010000000000?10????00000010?00??200000001010?100?
000000000111000000000000?0??00???????000000100000?
00000000030000100001002000000011100100000110 00?00110000100000000000000000000000000?0?
00100011000000001100?00?11101102?1120000?0010????0000?000?00??0000000??0?0????000000010000?
0???????????????20000?0?????0101?00?0?0010000?00000000000000010000???01000??0000000?
000000000200000000000000000000000?00100?0000020?20?000?00??0001000???????000000000000112010?1000?
00?1000001010000?003??0000??0??00?10001000001100010??01?1000?00000000000000?1000011?000000?
00000?????????????020?01000100000000000????0?000000010???????010100???????000010000210000?0?
0000010001000000000000001000000?0110000000100000?0000000?0001100000100?010010?
10001000000000000010000000000?0010010000000000000000??00000000000000010100???1?0011?1001??
1000001??00?0?0?
Sinotyrannus
???????????????????0?01???101?????????????????????????????????00??0?1?????????0?
0000101??????????????0???0??00??0?0?
000???0??0?????00?????????1?0000?0??100??0?????
11????????00?001??????????????????????????????
0???
00?000?0?0??0???????????00??????01?2?1??
10???0??1?00????
1???
?????????????????????0???????????????????????????0??
0001??????????????????????????????0?0??????0?????????????0?1????????????????????????????
0??00???????????
Proceratosaurus_bradleyi

?00?2???????????????00011?01112000?00??????????00?00???????000?00000010?00?

120000000101011???

?????????????????????????????????????00??0????0?0??0???????????110000?0?1100?00?11?0?1?2???????

0000010000????????00???0??00??10?0????

00???

???0???????0??????????????????00?0010112?10?1000?00?

1?0000101???0????????????????0?10001000001100001???????0???0???00000000??0?0?????????????0000??

000?????0??0?0??????00100?????0001???10?00000001000?????

11000???0?

00????????????1????0?0?????????????????????????0??0010???1?0?

1?????????????????000??????????

Dilong_paradoxus

?0002??000000???0?100011??1112000?00110?0000100000000100???????00000000?00??200000001010?1?0000?

0001?0??1000?????0??0?0?0??0???????0000001100?????0000000?00??1??00110201?0?0001?00?000?0??00??

00010000100?0000?00000?0?00000?000?0?00100010000000001100?001111?110201?20?000000????00000???0?

0???000001011000???000??00??00????0??????????????20000?0?0?0?0101000?0?0010000?0000000000000?0??

0????1000??0000000???0?000002000000000000000000??00?0?1000?0000?0?20?00???010000100????????

00000000010002?11?1000100?0?0000000000000103??0100?00??00010111100001100010??00?1100??

000000000010????00000?000100????????????????????020?010?010?0????01001??10?10002?00?11?????

0111000000010000010000 1???????00000110001????10000?0001??000???01100000000000??010000000??

11000????1?01?01001?????00??00???00???00000?00100?00?0000010?0?000000????00??0?000?0001?100???1??

10112????0??0000??????????

Eotyrannus_lengi

????????????????????000120??12??0???110????????????0?????????0?000?0?0??1???2000000010101 2?00?????

01?0??1????????????????????????????00000010????????0?0??

0???00000?0?00???00?0????00?0?????0?????0?1?000?00??10??0121???111?0?211?0000?0????????????????0??

00?00100???00??0???0?0010?0??0????????????????200?0?0?000?0?0?0?0?0?0010000?0000000?

0000????????????00???????????????0????????????????????????????????0????????????????????????

1???????????0?00?????1?00101???????00?0?01????01000?1010??1??0?00?00????????????????

0??????????????????????01?1??0????????????????????001??????0?00??????????????????????000?

1002110100??????????2??00???00???00????0???????????00??011011?000????????????????????????????01??

00?00100?000?0???0??????1?0??0?00????1000??????0?00????????0000?00???0000100?00??0??????????

0000???0?1????0??????011??????1???0?1?10??0???

Juratyrant

??0?00?

0000001?10??00000??0??????????????????????????????????????00?00?010000110101100000 11?01???0??10????

0???????????????????????1????????00???0010???????????????????????????2????

11??0?00100?0?

0???00?000000000?0?00?

000??00?????????????1????????????????????????????1?

0??

???

0????????00?100011?00100?0?????????????????10000001100100 0?????1100001????????????????????0??01??

1??1?????000?????????????0??????????????0000?0???????????000000??000??0000??????????????1??1??????

11??0????1?????????

Xiongguanlong

?0002???00000?????1?000220???0000?001101000?21000000?0001??000????????????????00000?101??

2000000000000111000??0?12?01000???????????????????

0000110???????????????????0??????0?00????000?????????1?000000???00?0?1121?2102011???0??0000?00?

000??????00???0??000????????????00?

00100???00?

0000000??20000???????0?????????????????????????????0???????000100?????????????0010?10001????1?00?

01?0?01?0000100??0010?0100??0??0??0?????0???01000?0???0?0100???001?11110010????100?1?11??00?

100?????????????00100100001?1?????????????????????????????????2?000??

000000011000011????????????????????????111??001???????011101 2????????????????00??0????????????1??1?

010?10??????????????00??????000?0??00001???????????000000?????000000???????10????1?????????1???0?

0?01?????????

Dryptosaurus

?????????????????????????20??0?0?0??2???????0000101?

1?????????????????????????0???00???????????????????10???????40?0?????????????[01]?0?0?2????????101?

01000100010001000??2???0???????????0?0?????????0?0????????????????00?????????????????11????

0??????????????????????????????????????0??????????????0????????????????????????????????????0?0?0010000?

00??????????????????????????????????0????????0020000000000??000??

0???00?01?????????????????????????

0??

????????????????????????????????????212?1?1??

10??????????????????????1?0???012?1?0111?????1????0??????0??????????????0????????????????

000000??????????????????00?1???00?????????00??0000?00001????01???????????0?1??00?0?0?????0???

Appalachiosaurus

????????????????????0?0?201110??????120??????????????????????00000?000?0????????00001010?

2?????????????????????????0?0?00??000010120?10???10??

010001000100010000002?0?00??????0????0?????????0?00100?11000000?1???1?021??????????????000??0??????

000?????0???????????0000???0????????????

0??00????????

002000000000000?0000?000?00?????????????????????????????????????00?????1????????000?000?01?

010111?101??1?0020?01?10??0?0???

000000010100010?00?????????????????11?110??1?????????0??1?????2?0?????????????????????????

0??????????????????????????????1?01110???111111111021?0000????????????0?????0?0???00???????????

0000000???00??0?????0???????????????????00?0??00000001???0???????01?????1??00000??1???????

Bistahieversor

?1102?????????10?2200002?01110000?001201000?21100000??????????00000000?20??2?000000101012??

0??

0??????????????2???????10??00??00?0??0???????????1200000011?00?00213?0?112??????0000000?????????

10??0????00001???0?0????00???????????????????????????????????????

00???

??11??????????0???????????????????0?1011001000?1010101?01011101010101002010001001???10000??

00000020000211000010000??1111111?1?1001011001211111?01000?010011111??????11?111101?10111111100?

11212101100112?10??2?00????111?????????????????0111?1???????????????????????????011?????01101111?

120010000????????????1??1?0?0???????????????????????????0??

0??11001??????1??????????000?11???????

Albertosaurus_sacrophagus

?1102??000000?10022000022011100000000120100002110000?000100000000000000020??200000001010120????0?

10?001?100?0????000??0????0???????000000010000???041001031200110000001012001000110??0?00?1000??

0010000020000000100000000000?000?0?0010?0120000001 1000?00213101112102011000000000???00000100?00??

0000010010000??000000001 0000?0?0????????0000020000?00000?0001001?0?0010000?000000000000010??

0????000??00??00?0000000000020000000000000000000?00??0100000??020100?000??0??001100?0110?

000000101110110010000[01]1001[01]10010111010201110021 1[01]1[01]1101?

0010000000100010201002001100100011111011111111101011001111111000[01]000111[01]

[01]1111000010110111111110111111100111212111100?12?10?121011???????1?????????????????

1111102011111?102111111021110 1??1??1???1111111111220100000000??00000101?011010?100010?000??00?

000000100?000000??0?00??110??00000000??0000?000000000101100111010111????010000001 1???????

Gorgosaurus_libratus

?11020?0000001100220000220111000000001201000021100000100010000000000000020012000000001010120?

0000010000101000000?000000000000?

000010000000010000000004100103120011000000101200100011010010001000?

00010000020000000100000000000000000?0?

001000120000000110001002131011121020110000000000000000001000000??0000010?10000??00000000010000[01]0?

0????????0000020000?00000?0001001?0?0010000?0000000000000100001 0??1000??0000000?

00000000020000000000000000000000?001000000002010 0?000100??00110000110?

000000101110110010000[01]100101001011101020112002 1101[02]1?

01000100000001000102010020011001000111110111111111010110011111110001000111111 11??

00110110111111110111111100111212111100112?10?121011???????1????1101????1?0?

0111110201011111021111010211101?011?1?2?111111111?22010000000100000010110110 10?100010?

00001000000000010000000000000?0000110?000000000??0000?000000000101100111101011110??0?0000001100?

000?

Alioramus

?01020?000000110022 00?0?20111000000001201000021100000100010000000000000000200?20??000010101?

000000010000111000000?00?00?0??000??????????????????????????????120011000[01]001012?010??????

001000100??0?01000?02??000??10??000000?0?0000????010??110000000110001?0213101?12102????000?

000000000000100000??00000100?0000??00000?00100?0?

0??00?0???000??

2000000000000000000?0??0?1?0????0020110?00???0??0011000???????0????00111?2????001110111110?

0111010100120121001021110101110001000010200001000000101101112011111111001011001111111?

10111001101110100011011011110110111011110011121211111 0??10000???000001000011110?

1012210111 11?????????????????1111010??????1?1111121??00?111?12?0100000??????0???0?1?1?010??

000????????????0????01?0000?000000?0????????????0000??0??00???0?000?1??1101111?1?1??1011??0?000?

11???????

Teratophoneus

?1?0???0???????0??2??????0111?00????1201???????0000???????????00??000?????????000010101??

1??0?????????????????????????????????

0??????????????????????????0????0????????????????1?0000?01?????????3?????????0???000????????????

10??0???00?001???0?????0??

1???

100??0????0?01???????????????2?021?0001??10???????????0???01?

1??????0???01??1?1??21?111?1001111101211?????????????????????????011?1?120?1????1??1100?

11?????????11????????10????????????1?????????????????1?01???1?????????????????????011?1?

1???????????????000??????????????????0?0?????????????????????????????0??

0???1??????1??????????00??1????????

Daspletosaurus

?11020?000000?

10022000022010100000001201000021100000100010000000000000020012000000010101201000001000001?100?

0????000??0????0???????000000010000???1410010312001100000010120010001 10??0100?1000??

001000000200000001000000000000000000?0?0010?00200000011000101213101112102011000000000???00000100?

00???00001001000O??000000000100000?0?0????????00000200000?00000?0001001?0?0010000?000000000000010??

0????1000??000??00?00000000020000000000000000000?00??01000000?020110?000??0??00110000110?

0000001011101200110201100211102111010210120121O101[12]11111111111110110102000021100101l[01]11211

212111111100?1110122111111011100110111l[01]100111110111101111111111110011122211110111 2?

100121011000111011110211111??????111111?211?111?112111??1??111?1??1????2?11????11?12???00000??

11000????1??1?010?1000???000?1000?0??????0?000000000???0011000?0????????????????????????

111011110??1??????????000?11???????

Tyrannosaurus_rex

?12020?

0110001100220000220101000000012010000211000001000100000000000000200120000000101012010000010000101

0000001000000000000?0????00000000010000???

1410010312001100000010120010001101001000100010001000002000000010000000000000000?0?

001000020000002100010121310121210201100000000000000000100000??000001001000O??0000000010000[01]0?

0????????000002000 0?000000000001001?0?0010000?000000000000010000????1000??000??00?

0000000002000000000000000000000000?0010000000020100?000?00??00110000110?

000000111111120011021 01[01]0[13]0110211110102111201 0??

10121011111011[01]110111102101021100101111121 1[12]12111111100111121230111111111110110111111111111

10110201111111111111121122212110111211111211211111101111102111121111111111111021101111112111111 0211

11111122112111111111111221100000001000000101101101 0?1000100000??00000000011000000000000100001100?

10000000??000000000000000101110111101011110110100000011001000 0

Tarbosaurus_baatar

??2020?011000?

1002200002201010000000120100002110000010001000000000000000200120000000101012010000010000101000 00?

1000000000000???????000000010000?

00141001031200110000001012001000110100100010001000100000200000001000000000000000?0?

001000020000002100010121310121210201100000000000000000100000??00000100100000??000000000010000?0?

0?????????????20000?00000?0001001?0?0010000?00000000000000010000????1000??0000000?000000000200000?

00000000000000?00100000000020100?000?00??00110000??????

000011111112001102101113011021111010210120010??

10121111111011111011110210102110010111112112121111111000111212301111111111101101111111111111101102

011111111111112112222212?1011121111121121111111?111?1021112??1??1?11111102110111?

112111111021111111122112111111111111221100000001000000101101101010?1000?

000000100000000001100000000000001000011 0??10000000??000000000000000101110111101011101 1??00000011????

0??

Halszkaraptor_escuilliei

?0010?????0????????000?0??110?11??002?12100101??0?1??10???????00??0100?[01]0??0?000210??1?00001?

0??0?001??000?[12]110?01?010?1? 1???0????????????0??0?????000??0?2?11???1???????????????????0???

100?0???0?????1101300010???000??00?0?????????????0?0?0?00001?00?0??? 00?00?[01]?1020020?00?

0??????????0??00???????0?????????0?000000?000?0??????????????????????????????????0?????????

0????????????????????????00???????00??????0?0?1?????1??????????0??00?00?010??001000????????

01??????1?0??????????00?00100010?000???? 10????0?0?0100?????0?????????????0??0???0???????0000?

0?????????00?????????00??1?0????0000??00?0?0????????????????????0?0???????????0??? 00000??

0001????????0????????0??000?????????00????????1????100??????1???1?????00?????0????????0????????0??????0?

0???000?0?1???00?01?? 00????110?0??0?????000?0?????????0?2?010010?000?0????????01?

0????????????????????001000?00?00010???????????1000???00?0?0?

Mahakala_omnogovae

???????????????101??????????????????????????1?00????????0?

0011?????????????????????????02?????1???00??0?11?011?211??1? 100011012210?????????????????00??1110000?

100?211101?2???????????????????0111100????0?011001101?00000??00??11?0?????0000????? 00????????

0????????????0????1[01]2?0??????0?????????????????0??1?00???????????

0000010000[01]0?????????????????????????????10??[01]0??? 0?0000???0?????????????100????0??0???

000000??0??001??0??1000000000?00000000?001??00?000????1???0??????011?0????????

000000????????????????????0???0??

0?????000?00????????0??????????????? 00010??????????????????????0????????????????????0000??????0?

01?000??????000??????????00????00100000???????0100000?0?000?100?0?000? 0????0?0?0???0????1?10???

00???????????0????010?20????0?0??0???????000000000??0??0100000000010?0????1?000???10????01????0??

00???0

Graciliraptor_lujiatunensis

???020???

1???????????????????????????0???11?12?????????????????01011?

1000001???????????????????????????????????????0??0?010?[01]11?1??0??0??00?????????????

0???????????????????00???????????????

200??0??????

10?0???000??0????????????10?0000??00?000??

10????????????????????????????????

000???

???

0???????????????????????????????????1????00?00?????????????????????????????0000????00??????100??0??

0???????0?????????00?0?00?????01???????????????????0???00?????????????????0?000?10?????????0?

0????????????????????????

Shanag_ashile

???????????????????1??1010110???????????????????????????????????00??011??????????

01001011??

???????????????????????????????????0????0?????????????????21100001???00???????????????????00??

0???

??

????????????????????????1?10???00?0?

000000??

????????????????????????0??00??00????????????????????

10??0?

0????????????????????0?????????????????????????????00???

1?????????????????00???????????

Epidendrosaurus

???0??1????????????????10??????????01?????0???????0??????

1??????????????????????????????????001??1??0??????001???????????????????????????????????????

0????????0000030?0?0???0?????1???????????????1?????????????????????????????3?

0??0???????0???????????????????0??0?0??????1???0??????????

0???0???0????????????????0??????000??????????????????????????100?00000??0?0??0?010????????

00??????1???????????0??

00??

???0????

0???

??0011??????0????????????????????????????0?????0?0???00?????????0?00??

0???????????????????????????????0?????????????0????????0????????????????????0010??????????

0?????????????????00?0?1?
